# Calcified apoptotic vesicles from PROCR^+^ fibroblasts initiate heterotopic ossification

**DOI:** 10.1002/jev2.12425

**Published:** 2024-04-09

**Authors:** Jianfei Yan, Bo Gao, Chenyu Wang, Weicheng Lu, Wenpin Qin, Xiaoxiao Han, Yingying Liu, Tao Li, Zhenxing Guo, Tao Ye, Qianqian Wan, Haoqing Xu, Junjun Kang, Naining Lu, Changhe Gao, Zixuan Qin, Chi Yang, Jisi Zheng, Pei Shen, Lina Niu, Weiguo Zou, Kai Jiao

**Affiliations:** ^1^ Department of Stomatology Tangdu hospital & State Key Laboratory of Oral and Maxillofacial Reconstruction and Regeneration & School of Stomatology, The Fourth Military Medical University Xi'an Shaanxi China; ^2^ Institute of Orthopaedic Surgery Xijing Hospital, Fourth Military Medical University Xi'an Shaanxi China; ^3^ State Key Laboratory of Oral and Maxillofacial Reconstruction and Regeneration & National Clinical Research Center for Oral Diseases & Shaanxi Key Laboratory of Stomatology, School of Stomatology The Fourth Military Medical University Xi'an Shaanxi China; ^4^ Department of Neurobiology The Fourth Military Medical University Xi'an Shaanxi China; ^5^ Center for Spintronics and Quantum Systems, State Key Laboratory for Mechanical Behavior of Materials, Department of Materials Science and Engineering Xi'an Jiaotong University Xi'an Shaanxi China; ^6^ College of Life Science Northwest University Xi'an Shaanxi China; ^7^ Department of Oral Surgery Ninth People's Hospital, Shanghai Jiao Tong University School of Medicine, Shanghai Key Laboratory of Stomatology, Shanghai Research Institute of Stomatology, and National Clinical Research Center of Stomatology Shanghai China; ^8^ State Key Laboratory of Cell Biology, CAS Center for Excellence in Molecular Cell Sciences, Shanghai Institute of Biochemistry and Cell Biology Chinese Academy of Sciences, University of Chinese Academy of Sciences Shanghai China

**Keywords:** calcified apoptotic vesicles, extracellular matrix, heterotopic ossification, macrophage polarization, osteogenic microenvironment

## Abstract

Heterotopic ossification (HO) comprises the abnormal formation of ectopic bone in extraskeletal soft tissue. The factors that initiate HO remain elusive. Herein, we found that calcified apoptotic vesicles (apoVs) led to increased calcification and stiffness of tendon extracellular matrix (ECM), which initiated M2 macrophage polarization and HO progression. Specifically, single‐cell transcriptome analyses of different stages of HO revealed that calcified apoVs were primarily secreted by a PROCR^+^ fibroblast population. In addition, calcified apoVs enriched calcium by annexin channels, absorbed to collagen I via electrostatic interaction, and aggregated to produce calcifying nodules in the ECM, leading to tendon calcification and stiffening. More importantly, apoV‐releasing inhibition or macrophage deletion both successfully reversed HO development. Thus, we are the first to identify calcified apoVs from PROCR^+^ fibroblasts as the initiating factor of HO, and might serve as the therapeutic target for inhibiting pathological calcification.

## Graphical abstract

Calcified apoVs increased calcification and stiffness of tendon extracellular matrix, and initiated the pro‐osteogenic microenvironment during HO development. PROCR^+^ fibroblasts released calcified apoptotic vesicles (apoVs) in the early stage of HO and calcium was continuously enriched in calcified apoVs via Annexin channels. When the crystallites were produced within the calcified apoVs, the mechanical stress within the calcified apoVs resulted in the puncture of the membrane and the release of crystallites. The increased stiffness of ECM caused by calcium and phosphorus deposition promoted M2 macrophage polarization, which initiated the osteogenic cascade in HO.

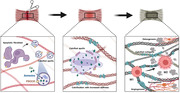

## 1 INTRODUCTION

Heterotopic ossification (HO) comprises the abnormal formation of ectopic bone in extraskeletal soft tissue (Agarwal et al., 2017), which causes significant pain, swelling, joint stiffness and gradual limitation of movement, finally leading to disability (Edwards & Clasper, 2015). Current treatment strategies for HO comprise nonsteroidal anti‐inflammatory drugs, bisphosphonates and surgical resection in severe cases (Crupi et al., 2020; Ranganathan et al., 2015). These treatments, however, have a low cure rate and a high recurrence rate, resulting in a large financial burden for patients (Meyers et al., 2019). The bottleneck lies in the limited understanding of the cytological basis and microenvironmental changes in HO, which are determined by the abnormal cell fate of resident cells, including immune cells, fibroblasts and so on (Wang et al., 2018). Significantly, the changes of physical and chemical properties of the tendon microenvironment, such as calcium and phosphorus deposition and tendon stiffness (Best et al., 1993; Isaacson et al., 2011), have also been shown to relate to the progression of HO. However, the research on the causative factors and mechanisms of HO at early stage is still insufficiently advanced.

Our previous studies suggested that osteoarthritis (OA) was initiated by pathological calcification, and calcification expedited inflammation and accelerated disease progression (Sun et al., 2020). Crystallite‐induced stress results in articular chondrocyte phenotype changes and induces the production of pro‐inflammatory and catabolic mediators. The crystallites also cause a stiffness imbalance in the cartilage, aggravating its biomechanical properties and promoting the manifestation of osteoarthritis (OA) chondrocyte phenotypes (Yan et al., 2020). This explains why cartilage degradation progresses rapidly once calcification exists. Extracellular vesicles (EVs) are a heterogeneous population enveloped by a plasma membrane that have been implicated in the bone formation and mineral apposition, termed calcified EVs (Anderson, 1969; Aikawa & Blaser, 2021; Chen et al., 2018; Chistiakov et al., 2017; Durham et al., 2018; Golub, 2011; Strzelecka‐Kiliszek et al., 2017). We demonstrated that autophagy‐derived microtubule‐associated proteins 1A/1B light chain 3B (LC3)‐positive calcified EVs from autophagosomes initiated pathological cartilage calcification in OA (Yan et al., 2022). A series of studies have revealed that cells undergoing apoptosis can produce heterogeneous apoptotic vesicles (apoVs), and apoVs are revealed unique biological and functional characteristics that are emerging as crucial regulators for diverse processes (Durham et al., 2018). Notably, accumulating evidences showed that apoptosis was involved in calcification‐related diseases (Hashimoto et al., 1998). Apoptosis precedes vascular smooth muscle cell calcification and inhibiting apoptosis also inhibits calcification in atherosclerosis and hypertension (Proudfoot et al., 2000). However, the feature, fate and function of apoVs in pathological calcification need to be explored.

Herein, we performed single cell sequencing of tendon cells from different stages of HO combined with scanning electron microscopy (SEM), transmission electron microscopy (TEM), cryogenic‐electron microscopy (cryo‐EM) and atomic force microscopy (AFM) to clarify the stage‐specific and location‐specific pathology of minerals in HO tendons. Based on this model, we identified calcified apoVs in HO tendon and investigated their origin and contribution to pathological calcification. Calcified apoVs initiated pathological calcification in HO and the calcification‐induced increased stiffness promoted the formation of a local osteogenic microenvironment. Our results revealed a new pathomechanism of HO initiation and identified calcified apoVs as a potential treatment target for HO.

## 2 MATERIALS AND METHODS

The group designation and experimental scheme are described in Figure S1. The detailed method for Micro‐CT analyses, histologic and immunohistochemistry evaluation, SEM, elemental mapping, calcein fluorescent labelling and histomorphometrical analysis, fourier transform infrared spectroscopy, single‐cell RNA‐sequencing (scRNA‐seq) analysis, cell culture, PDMS substrates and live‐cell imaging are included in the supplementary information.

### 2.1 Rat Achilles tenotomy model and treatment

In vivo experiments were conducted with male Sprague‐Dawley rats, aged 8 weeks, weighing 200–300 g, obtained from the Fourth Military Medical University's Laboratory Animal Research Center. Fourth Military Medical University's Institutional Animal Care and Use Committee approved all surgical procedures for those experiments (ethics approval number: 20220906). To construct the rat model of achillotenotomy, rats were deeply anesthetized and the Achilles tendon of HO group was transected along the midpoint completely. All incisions were closed with interrupted 4‐0 silk sutures. Rats in sham group suffered from the same type of skin incision but not achillotenotomy. All rats received analgesics to relieve pain after surgery and were allowed to move freely in their cages. During the entire experimental period, none of the rats died and there was no difference between the right and left legs in the occurrence of ectopic bone formation.

### 2.2 Cryogenic‐electron microscopy (cryo‐EM)

Quantifoil Jena R2/2/gold grids coated with holey‐carbon (2 µm hole size) supporting film (Electron Microscopy Sciences, Hatfield, PA, USA) were plasma‐treated and used within 30 min after treatment. Sections (90 nm thick) of tendon tissues were obtained and dropped on the surface of the grids. After vitrification (∼1000 Å thick), the grids were transferred to a Gatan 626 cryo‐transfer holder and maintained at a temperature below  −170°C during cryo‐EM observation with a Talos F200C microscope (FEI, Hillsboro, OR, USA) at 100 kV. The electron dose for each exposure was 20e Å^−2^. Selected area electron diffraction was performed to confirm the crystallinity of the minerals in tendon.

### 2.3 Atomic force microscope

Atomic force microscope (Asylum Research, Santa Barbara, CA, USA) was used to analyze 15 µm sections of the tendon tissue from different groups. Micromorphological imaging of the tendon tissue was performed using a silicon probe (PPP‐NCLR‐20, Nanosensors, Neuchatel, Switzerland) with a force constant of 42 N/m and a resonance frequency of 161 kHz after the hydrated sections were naturally dried. All measurements were repeated for three positions of each tissue sample and the values were averaged.

### 2.4 Isolation of the extracellular vesicles (EVs) from the tendon

To collect the EVs from the tendons of the sham and HO groups, tendons were harvested and washed with PBS on ice. The tendon was sliced and incubated with collagenase D and DNase I (both MilliporeSigma) for 120 min at 37°C. A filtration step through a 0.70 µm pore‐size filter was applied to remove the largest elements. The remaining liquid was differentially centrifuged at 800 × *g* for 10 min and 2000 × *g* for 30 min to remove cells and tissue debris. The supernatant was then further centrifuged at 7000 × *g* for 30 min to collect the EVs, which were washed in PBS using the same centrifugation conditions (Crescitelli et al., 2021).

The pellets of EVs were resuspended in 400 µL of PBS for nanoparticle tracking analysis, TEM and flow cytometry, or functional assay of mineralization. The protein content of the isolated EVs was measured through a BCA Kit (Beyotime BioTech, Jiangsu, China).

### 2.5 Nanoparticle‐tracking analyses

The size distribution of the EVs was evaluated using the ZetaView PMX 110 (Particle Metrix, Meerbusch, Germany) equipped with a 405 nm laser. The EVs collected from the supernatant or tendons were resuspended in filtered PBS to achieve a final concentration between 1 × 10^7^/mL and 1 × 10^9^/mL. A video of 60‐s duration was taken with a frame rate of 30 frames/sec, and particle movement was analysed using NTA software (ZetaView 8.02.28).

### 2.6 Flow cytometry analysis

For flow cytometry analysis of tendon cells, tendon tissues were harvested and minced with scissors, then enzymatically digested in CO_2_‐independent incubator shaker (Kuhner, ISF1‐XC) with 1 mg/mL Collagenase I (Sigma‐Aldrich, SCR103) and IV mixture (Sigma‐Aldrich, C4‐28‐100MG) for 3 h at 37°C. After diluted with serum‐free medium and centrifuged at 200 × *g* for 10 min, the cell pellets were resuspended in ACK lysis buffer (ThermoFisher, NC9067514) to remove blood cells. Prior to staining, the cells suspended in FACS buffer comprised of PBS with 5% BSA (Sigma‐Aldrich, SRE0096) were filtered through a 40 µm mesh. Dissociated single cells were stained with anti‐PROCR (bs‐9506R, Bioss, Beijing, China) and anti‐rabbit IgG (H+L) APC‐conjugated secondary antibodies (SA00014‐9, Proteintech, Wuhan, China). The detection was performed using a flow cytometer (CytoFLEX, Beckman Coulter, Brea, CA). The flow cytometric analysis was performed with FlowJo 10.0 software (Flow Jo LLC, Ashland, OR, USA) after washing twice by centrifugation at 600 × *g* for 5 min with ice‐cold DPBS + 2% FBS.

The EVs re‐suspended in PBS were incubated with Annexin V‐PE (Annexin V‐PE Apoptosis Detection Kit, MedChemExpress, NJ, USA) and the Ca^2+^ marker (Fluo‐4, AM ester, US Everbright, Suzhou, China) and mixed for 30 min. After washing the EVs 3 times with 2% BSA in PBS, Annexin V and Ca^2+^ detection was performed using a flow cytometer (CytoFLEX, Beckman Coulter, Brea, CA). The forward scatter of the device was chosen to be 100, and the flow cytometry analysis was performed to differentiate the EVs by the logarithmic scale used for forward and side scatters (Zhang et al., 2022). Apoptosis in fibroblasts cultured in vitro was assessed using Annexin V Apoptosis detection kit (Annexin V‐PE, Propidium Iodide (PI) solution and Annexin V binding buffer). FACS analysis of the fibroblasts that are in early (annexin V+/PI2) or late (annexin V+/PI+) apoptotic phase was performed using the FlowJo 10.0 software (Flow Jo LLC, Ashland).

### 2.7 3D self‐assembled collagen fibrils

An acetic acid/collagen stock solution (5 mg/mL) derived from rat tail tendon was used for 3D self‐assembled collagen fibrils (Shen, Jiao, et al., 2022). The self‐assembled collagen solution was dropped on a 400 mesh Au TEM grid and dried at room temperature. The grids were rinsed with deionized water three times and air‐dried. For the cell assay, room‐temperature drying was also performed on the collagen solution (Shen, Wang, et al., 2022).

### 2.8 In vivo pathological collagen mineralization models

The intratendon ectopic calcification model was created in rats (*n* = 3). The apoVs were seeded into 3D collagen scaffolds (ACE Surgical Supply Co., Brockton, MA, USA) under sterile conditions. The 3D collagen scaffolds (5 mm × 5 mm) were implanted intratendonly into separate pockets in 6‐week‐old rats. After 1 and 3 weeks, the rats were sacrificed and their Achilles tendons were collected for subsequent analysis.

### 2.9 ApoVs induced the calcification of the collagen

The collagen solution was dropped on a 400‐mesh nickel TEM grid and air‐dried. The collagen‐coated grids were floated upside down over solutions of apoVs (250 µg/mL) from different groups and imaged using TEM.

### 2.10 Transmission electron microscope (TEM)

The tendons and tendon‐containing collagen hydrogels were fixed in 2.5% glutaraldehyde in phosphate buffer (0.01 M, pH = 7.4). The specimens were post‐fixed in 1% osmium tetroxide, dehydrated in an ascending series of ethanol, immersed in propylene oxide and embedded in epoxy resin. Ninety nanometre‐thick sections were obtained, stained with uranyl acetate and lead citrate and observed using a JEM‐123 transmission electron microscopy (TEM, JEOL, Tokyo, Japan) at 110 kV.

For TEM observation of EVs, the EV pellet was re‐suspended in 200 µL of PBS and 20 µL of EVs was deposited on 200‐mesh formvar‐coated copper grids and dried at room temperature. The EVs were negatively‐stained with uranyl acetate prior to imaging.

### 2.11 Mass spectrometry analysis of apoVs

After isolation, the membrane protein of the apoVs were extracted with Membrane and Cytosol Protein Extraction Kit (20127ES50, Yeasen, China) and then treated with Radioimmunoprecipitation assay lysis buffer. Then, the mixture was injected into the mass spectrometer (Orbitrap Eclipse Tribrid Mass Spectrometer, Thermo Fisher Scientific, Waltham, MA) after trypsin digestion. After calibrating using the standard compounds, the mass spectrometer was operated in the data‐dependent mode. In this mode, the mass spectrometer cycled between full MS scans with m/z 100−1800. Only proteins with high protein false discovery rate confidence were considered for further analysis.

### 2.12 Calcium channel blockers

We employed the small Annexin A2 inhibitor molecule, LCKLSL hydrochloride (MedChemExpress), to study the function of Annexins in the calcified apoVs (Liu et al., 2023). The groups were control group and LCKLSL hydrochloride treatment group (5 µmol/L LCKLSL). After the treatment for 3 days, the samples were collected and used for fluorescence microscopy (Ca staining) and TEM detection.

### 2.13 Molecular dynamic simulation

Molecular dynamic simulations were conducted between DPPC (Di Palmitoyl Phosphatidyl Choline)/DPPS (Di Palmitoyl Phosphatidyl Serine) bilayers (DPPC:DPPS = 1:1) with the triple‐helix region of collagen type I (PDB ID:7CWK) using the GROMACS 2021.5 package (Abraham et al., 2015). Lipid and protein were parameterized by Lipid21 (Dickson et al., 2022) and Amberff14sb (Maier et al., 2015) force field. An initial structure with a 12 × 12 × 15 nm^3^ cubic box of lipid bilayer containing 234 DPPC and 234 DPPS was constructed at the CHARMM‐GUI website and collagen was 1.4 nm away from the bilayer, then system was dissolved in TIP3P water (transferable intermolecular potential with three points; 48717 water molecules) and 231 Na^+^ were added to maintain electrical neutrality. Energy minimization was performed using the steepest descent algorithm with a force tolerance of 500 kJ/mol/nm. Periodic boundary conditions were imposed in all three directions. Then, these systems were relaxed for 1 ns under NPT MD simulations, and position restraints with a constant of 1000 kJ/mol/nm in three directions were performed on heavy atoms of lipids and proteins (Bussi et al., 2007).

Next, 200 ns NPT MD simulations were performed on the bilayer‐collagen system. Pressure was maintained at 1 bar using a Parrinello‐Rahman barostat in a semiisotropic manner (xy and z directions) and the temperature was maintained at 310 K using a V‐rescal thermostat (Nosé & Klein, 1983; Wang et al., 2004). The LINCS algorithm was performed for the constrained bond lengths of hydrogen atoms. Lennard‐Jones interactions were calculated within a cutoff of 1.2 nm, and electrostatic interactions beyond 1.2 nm were treated using the particle‐mesh Ewald (PME) method with a grid spacing of 0.16 nm. UCSF ChimeraX (Pettersen et al., 2021) was used to visualize the results.

### 2.14 Quantitative real‐time reverse transcription polymerase chain reaction (qRT‐PCR)

To investigate the influence of macrophages on the formation of HO, gene expression levels of *iNOS*, *IL‐1β* (encoding interleukin‐1 beta), *TNF‐α* (encoding tumour necrosis factor alpha), *CD206*, *FIZZ1*, *Arg1* (encoding Arginase 1), *netrin 1*, *slit 3*, *netrin 3*, *VEGF*, *CD31*, *Emcn* of the macrophages were evaluated using qRT‐PCR. According to the manufacturer's instructions. *Gapdh* (encoding glyceraldehyde‐3‐phosphate dehydrogenase) was used as the housekeeping gene. Total RNA of the specimens was extracted with Tripure Isolation Reagent (Life Technologies) and quantified by ultraviolet spectroscopy at assigned time points post‐induction. The concentration and purity of the extracted RNA were determined by measuring the absorbance at 260 and 280 nm (BioTek, Winooski, VT). cDNA was synthesized using a PrimeScript RT reagent kit (Takara Bio Inc., Shiga, Japan). The quantitative real‐time polymerase chain reaction was performed using the cDNA as the template on a 7500 Real Time PCR System (Applied Biosystems, Foster City, CA). Reverse transcribed product (1 µL) and 1× SYBR green (Molecular Probes, Eugene, OR) were included in 25 µL reaction mixture (10 mM Tris–HCl, pH 8.3, 50 mM KCl, 1.5 mM MgCl_2_, 200 µM of dNTP mix, 0.2 µM of each primer and 1 unit of Taq DNA polymerase). Oligonucleotide primers were designed using Oligo 6 primer analysis software. Each cycle consisted of 1‐min denaturation at 94°C, 1 min annealing at 58°C and 1 min extension at 72°C. Results obtained after calibration using the *Gapdh* expression level were calculated using the 2^−ΔΔCt^ method and presented as fold increases relative to the non‐stimulated control (technical replicates *n* = 3 for each group). All the primer sequences were listed in Table S1.

### 2.15 Z‐VAD‐FMK injection

To inhibit fibroblast apoptosis in the Achilles tendon, Z‐VAD‐FMK (T7020, TargetMol) dissolved in normal saline was injected into rats at 1 µg/rat, three times a week. An equal volume of normal saline was injected into the sham group.

### 2.16 Macrophages depletion

For macrophage depletion, the injured rats were injected intravenously with clodronate‐liposomes (5 µL/g) immediately after Achilles tenotomy and every 3 days for 6 weeks post‐surgery. The control group received an equivalent volume of PBS‐loaded liposomes under the same conditions.

### 2.17 Statistical analyses

Statistical analyses were performed using GraphPad Prism 8.0 (GraphPad Software, San Diego, CA). All data are expressed as the mean ± standard deviation. In order to test the normality and homoscedasticity assumptions of the corresponding data sets, the Shapiro‐Wilk test and modified Leven test were used. The differences between groups were evaluated using the Student's *t* test, one‐factor or two‐factor analysis of variance (ANOVA) followed by Holm‐Šidák multiple comparison tests. To ensure the validity of the observations, quantitative experiments were repeated at least 3 times. For all tests, statistical significance was present at *α* = 0.05.

## 3 RESULTS

### 3.1 Tendon calcification leads to increased stiffness of matrix in the early stage of HO

**FIGURE 1:**
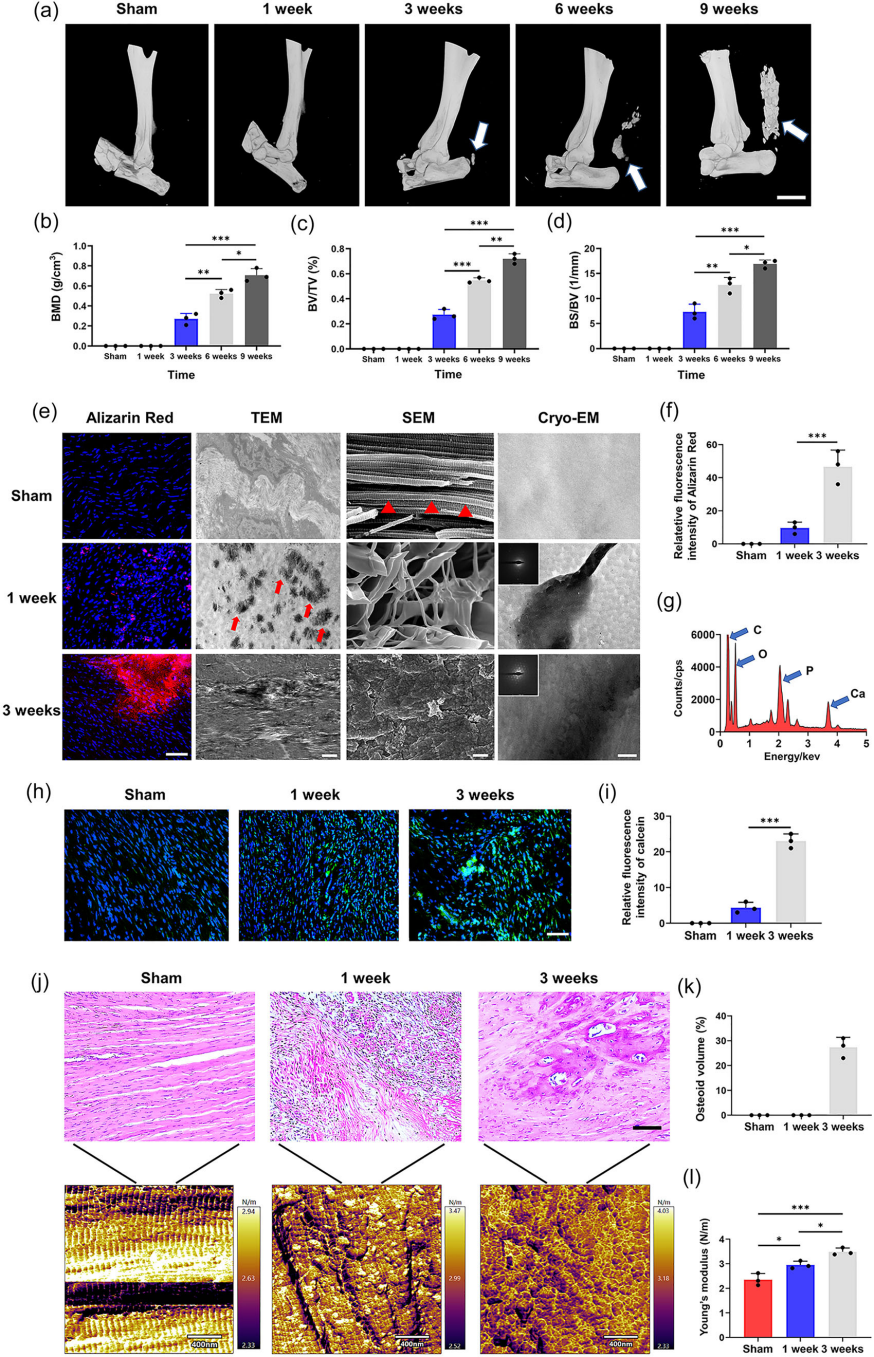
Calcification of the Achilles tendon leads to increased stiffness of the ECM in the early stage of HO. (a) Micro‐computed tomography (micro‐CT) images of Achilles tendons of rats from the sham and HO groups after 1, 3, 6 and 9 weeks. Heterotopic ossification was observed in the HO group (white arrows in a). Scale bar, 5 mm. (b–d) Quantitative analysis of bone histomorphometric parameters based on the micro‐CT images in (a): (b) bone mineral density (BMD), (c) bone volume/tissue volume (BV/TV) and (d) bone surface/bone volume (BS/BV). (e) Alizarin red S staining of Achilles tendons of rats from the sham and HO groups after 1 and 3 weeks. Areas stained with alizarin red S indicated calcified regions. Alizarin red S, red; DAPI, blue. Scale bar: 50 µm. Representative TEM (Scale bar: 1 µm), SEM (Scale bar: 500 nm), and Cryo‐EM (Scale bar: 200 nm) images of the Achilles tendons of rats from the sham and HO groups after 1 and 3 weeks. Calcifications were widely dispersed in the tendon ECM of 1 week HO group (red arrows) and collagen banding was only observed in sham group (red triangles). Electron diffraction of the mineral was inserted. (f) Quantitative analysis of the calcified regions stained with Alizarin red S in (e). (g) Elemental analysis of the tendon minerals in the 1 week HO group. (h) Fluorescent labelling observations of calcium of Achilles tendons of rats from the sham and HO groups using calcein. Scale bar: 50 µm. (i) Quantitative analysis of the calcified regions in (h). (j) Post‐AFM histological overview and representative AFM stiffness map of the Achilles tendons of rats from the sham and HO groups after 1 and 3 weeks. Young's modulus increased after tendon injury compared with that in the sham group. (k) Quantitative analysis of osteoid volume based on H&E staining images in (j). (l) Quantitative analysis of AFM stiffness based on (j). Data represent the means ± standard deviations (n = 3). Statistical analyses were performed using one‐way ANOVA with a post‐hoc Tukey's test. *p < 0.05, **p < 0.01, ***p < 0.001.

The pathogenesis of HO involves initial changes in various cellular components combined with increased stiffness of matrix, followed by progression of tendon osteogenesis and remodelling. Current treatment primarily focuses on the progression stages, while the initial pathogenic factor is unknown. Therefore, we systematically investigated HO at different stages, especially initiation, using the rat Achilles tendon calcification model. Micro‐computed tomography (CT) showed no radiographic evidence of bone formation at 1 week after Achilles tenotomy. Some ectopic bone nodules were identified in the Achilles tendons by 3 weeks. By 6 weeks, new bone (radio‐opaque zones) in the Achilles tendon region was detected in 60% of tendons and in 100% by 9 weeks (Figure 1a). Bone mineral density (BMD), bone volume/tissue volume (BV/TV), and bone surface/ bone volume (BS/BV) analyses identified increased bone mass at different times in the HO group (Figure 1b–d). Next, we identified tendon mineral distribution, morphology and composition at different HO stages. Macroscopically, alizarin red S staining indicated the presence of minerals at 1 week in HO group, which was increased at 3 weeks (Figure 1e,f). TEM and SEM showed thickened and calcified collagen fibrils and calcifications that were widely dispersed in the tendon ECM of 1 week HO group. At 3 weeks, collagen banding disappeared and abundant minerals were found on the surface of collagen fibrils (Figure 1e). These results indicated that HO development involves disordered tendon calcification. The chemical composition of these minerals was analyzed using energy‐dispersive X‐ray spectroscopy. There were significant variations in Ca and P contents in the 1 and 3 week HO samples (Ca/P ratio = 1.76 (1 week); 2.05 (3 weeks)) (Figure 1g and Figure S2). The minerals in the samples were also characterized using cryo‐EM. Selected area electron diffraction showed minerals along the collagen fibrils as early as 1 week. More extensive mineralization was identified after 3 weeks (Figure 1e). New bone formation and mineralization were indicated using calcein at different time points (Figure 1h and Figure S3). In the HO group at 3 weeks, the relatively fluorescence intensity of the calcein‐labelled area (green; mineralization) was significantly higher than that at 1 week, both of which were higher than that in the sham group (Figure 1i). Micro‐infrared analysis comparisons of the changes in collagen and the calcium phosphate content of Achilles tendon tissues in the sham and HO groups showed that in the HO group, the collagen content in the Achilles tendon decreased and the phosphate content increased. Compared with that at 1 week after injury, the phosphate content at 3 weeks was significantly upregulated (Figure S4).

To correlate the nanomechanical profiles with the pathohistological results in the sham and HO groups, AFM analyses of in vivo tendon tissues were performed. We also assigned individual stiffness characteristics to specific tissue morphologies (stained with haematoxylin & eosin (H&E)) to perform more detailed measurements within defined regions of the samples (Figure 1j), particularly in the early injury group. A map of stiffness values from the sham sample revealed an average stiffness of 2.63 N/m. In comparison, the calcified tendon from the 1 week HO group had an increased average stiffness of 2.99 N/m. The average stiffness of individual calcifications embedded in the ECM was further increased (3.47 N/m) and post‐AFM H&E staining confirmed inflammatory cell infiltration of the injury site without the formation of osteoid (Figure 1j, middle). The stiffness of a different areas of a representative sample from the 3 week HO group varied from 2.31 to 4.03 N/m, indicating marked mechanical heterogeneity across the sample. Post‐AFM H&E staining confirmed the extensive mineralization of the ECM in the 3 week HO group (Figure 1j, bottom right, and 1k). The correlation of local AFM data with matching histology corroborated that the stiffness peak represents typical calcification surrounded by softer ECM (Figure 1j,l and Figure S5). These results indicated that tendon calcification was accompanied by increased matrix stiffness, especially in the early stage of HO. However, the origin of pathological calcification and the cytological behaviour behind requires study.

### 3.2 Single‐cell RNA‐sequencing (scRNA‐seq) analysis identifies novel apoptotic‐preferential PROCR^+^ fibroblasts in the early stage of HO

**FIGURE 2:**
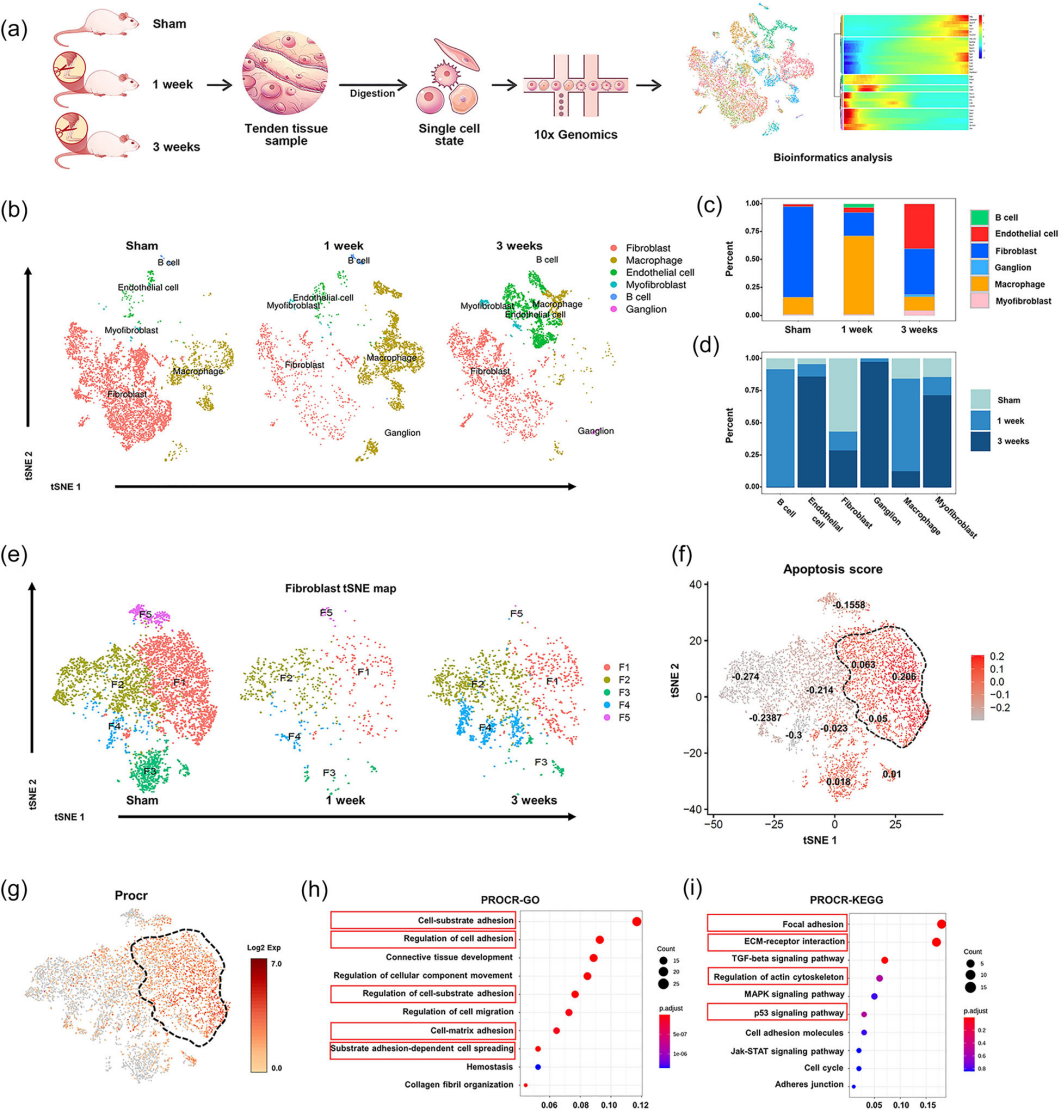
Single‐cell RNA‐sequencing (scRNA‐seq) analysis identifies novel apoptotic‐preferential PROCR^+^ fibroblasts in the early stage of HO. (a) The workflow depicting the collection and processing of specimens of sham and HO tendons for scRNA‐seq. (b) Dimension reduction presentation (via tSNE) of combined single‐cell transcriptome data from Achilles tendons of rats from the sham and HO groups after 1 and 3 weeks (*n* = 14402). Each dot represents a single cell and is labelled with corresponding cell categories and is coloured according to its cell type identity. Clusters were generated using a resolution of 0.2 before subclustering into major cell types according to the Methods. The Seurat 3 R‐Package segregation grouped the cells into 6 distinct cell clusters. (c and d) Quantitative analysis of clusters based on the combined single‐cell transcriptome data in (b). (e) tSNE of fibroblast clusters (F1–F5). (f) Apoptosis score analyses of fibroblasts based on (e). (g) Bioinformatic analysis of PROCR^+^ cell populations based on (e). (h) Representation analysis of GO categories showing different functions for PROCR^+^ cells. (i) Representation analysis of KEGG categories showing different functions for PROCR^+^ cells.

Fibroblasts are the principal cell type in ligament tissue and the core player in the initiation of tissue ossification (Yang et al., 2011; Zhang et al., 2013). However, the role and the underlying mechanism of fibroblast‐mediated HO initiation and progression are unclear; therefore, we systematically investigated the dynamic cellular changes in fibroblasts from different stages of HO using 10x Genomics scRNA‐seq (Gao et al., 2022). After unsupervised graph clustering of the three datasets combined (sham, 1 week and 3 weeks), the Seurat 3 R‐Package was used to segregate the captured cells into six distinct cell clusters, including two principal cell types according to the cell assemblies on the t‐distributed stochastic neighbour embedding (t‐SNE) plot: the fibroblast cluster and the macrophage cluster (Figure 2a,b). To annotate these populations, we plotted the fraction of cells expressing each marker across all stages using the differentially expressed genes (DEGs) among the cell types (from the heatmap in Figure S6). We investigated how the fractions of different cell clusters changed in the early stage of Achilles tendon injury. The number of fibroblasts decreased significantly after Achilles tendon injury after 1 week and 3 weeks (Figure 2c,d). To further investigate the mechanism underlying cellular activity related to the formation of early calcifications at the single‐cell level, we focused on fibroblasts. A t‐SNE plot from the scRNA‐seq analysis displayed 5 distinct fibroblast subpopulations with different gene expression patterns (Figure 2e). Extra‐skeletal mineralization is a consequence of apoptotic cell death or at least intersects with certain apoptotic signalling pathways and apoptosis is therefore a key factor in calcification (Hashimoto et al., 1998). Notably, apoptosis score analyses identified cluster 1 fibroblasts as the apoptotic‐preferential cell population (Figure 2f). The protein C receptor (PROCR) was especially active in the apoptosis‐related clusters (Wang et al., 2019), and immunostaining and flow cytometric analysis of tendon samples confirmed the presence of PROCR^+^ fibroblast subpopulations (Figure 2g and Figure S7). Gene ontology (GO) and Kyoto Encyclopedia of Genes and Genomes (KEGG) analyses also revealed that PROCR^+^ fibroblast subpopulation was mainly enriched in functions such as cell‐substrate adhesion, cell‐matrix adhesion and focal adhesion (Figure 2h,i and Figures S8 and S9). These data revealed that the tendon fibroblasts comprised heterogeneous subpopulations and PROCR^+^ fibroblasts were apoptotic preferential subpopulations in the early stage of HO.

### 3.3 Calcified apoVs from PROCR^+^ fibroblasts markedly aggregated and induced tendon calcification in the early stage of HO

**FIGURE 3:**
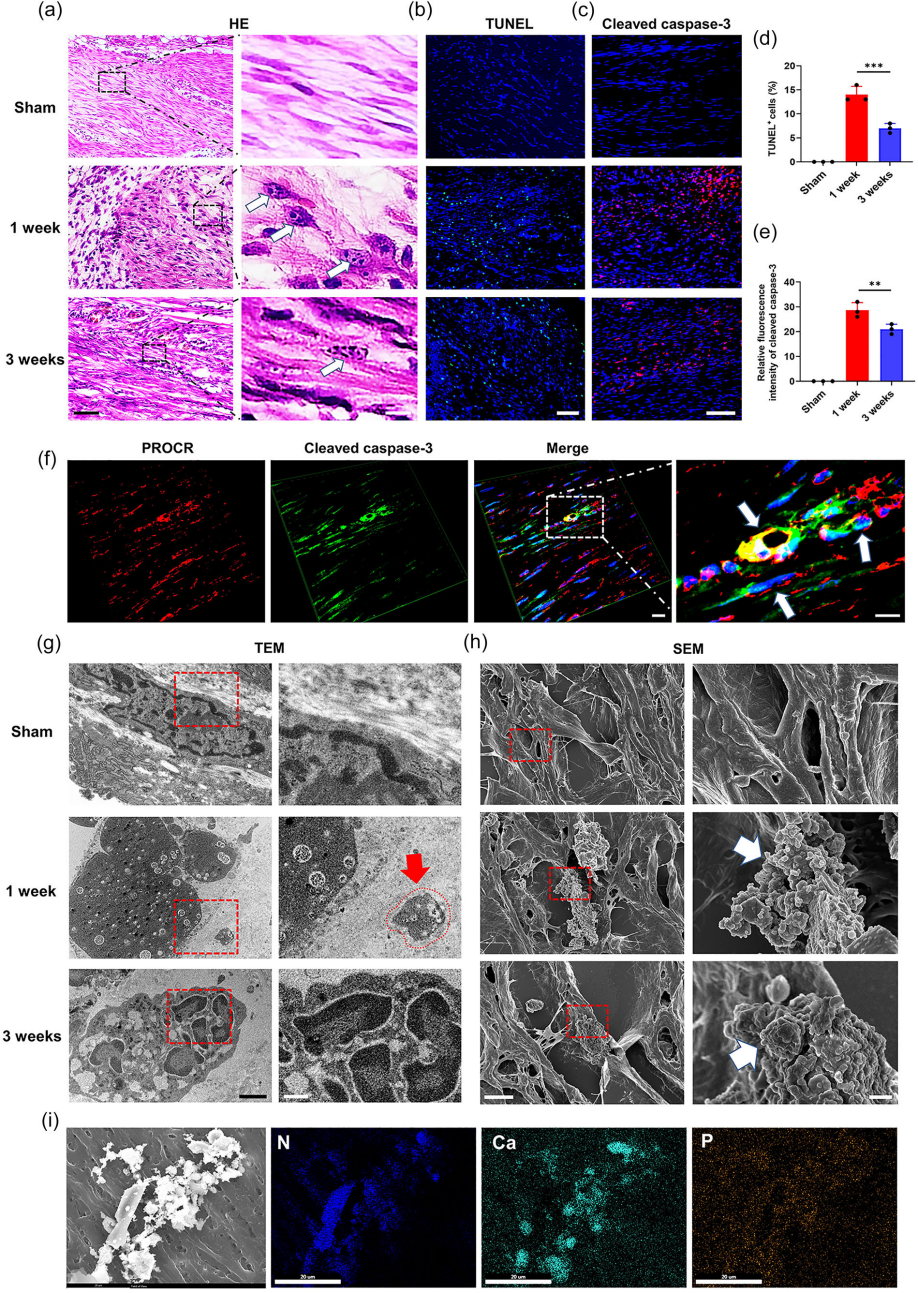
The calcified apoVs from PROCR^+^ fibroblast markedly aggregated and induced tendon calcification in the early stage of HO. (a) H&E staining of Achilles tendons of rats from the sham and HO groups after 1 and 3 weeks. Scale bar: 50 µm. High magnification of the black rectangle outline in the low magnification image showing apoptotic cells (arrows). Scale bar: 10 µm. (b) Representative images of TUNEL staining from the sham and HO groups after 1 and 3 weeks. Scale bar, 50 µm. (c) Representative immunofluorescence staining images of cleaved caspase‐3 from the sham and HO groups after 1 and 3 weeks. Scale bar, 50 µm. (d) Quantitative analysis of TUNEL^+^ cells in (b) (*n* = 3). (e) Quantitative analysis of the relative fluorescence intensity of cleaved caspase‐3 of Achilles tendons from sham and HO groups after 1 and 3 weeks (*n* = 3). RFI: relative fluorescence intensity. (f) Representative images of immunofluorescence staining of an Achilles tendon derived from the HO group after 1 week. PROCR, red; Cleaved caspase‐3, green; DAPI, blue. Scale bar, 10 µm. High magnification of the white rectangle outline in the low magnification image showing the apoptotic PROCR^+^ fibroblasts (arrows). Scale bar: 5 µm. (g) Representative TEM images of Achilles tendons of rats from the sham and HO groups after 1 and 3 weeks. Scale bar: 1 µm. High magnification of the red rectangle outline in the low magnification image showing the cells and calcified apoVs (arrows). Scale bar: 300 nm. (h) Representative SEM images of Achilles tendons of rats from sham and HO groups after 1 and 3 weeks. Scale bar: 1 µm. High magnification of the red rectangle outline in the low magnification image showing apoptotic cells (arrows). Scale bar: 500 nm. (i) SEM images and elemental mapping showing the aggregation of the calcified apoVs in the 1‐week HO group. Scale bar: 20 µm. Data represent the means ± standard deviations (n = 3). Statistical analyses were performed using one‐way ANOVA with a post‐hoc Tukey's test. **p < 0.01, ***p < 0.001.

Previously, we identified that calcified EVs initiated pathological calcification in OA (Yan et al., 2022); however, whether apoptotic PROCR^+^ fibroblasts release apoVs and directly induce HO initiation is unknown. H&E staining showed shrinkage of the nucleus and condensation and fragmentation of the chromatin in Achilles tendon fibroblasts in the HO group (Figure 3a). The percentage of tunnel‐positive fibroblasts was 14% at 1 week and 7% at 3 weeks in HO model (Figure 3b,d). Caspases execute apoptosis by selectively targeting and cleaving many key molecules in the cells. In the HO group, the relative fluorescence intensity of cleaved caspase‐3 was significantly increased during the inflammation phase at 1 week and decreased at 3 weeks (Figure 3c,e). Immunofluorescence staining for PROCR, also showed that the PROCR^+^ fibroblasts of 1 week HO group had abundant cleaved caspase‐3 (Figure 3f and Figure S10). Therefore, PROCR^+^ fibroblasts underwent apoptosis after Achilles tendon injury.

**FIGURE 4:**
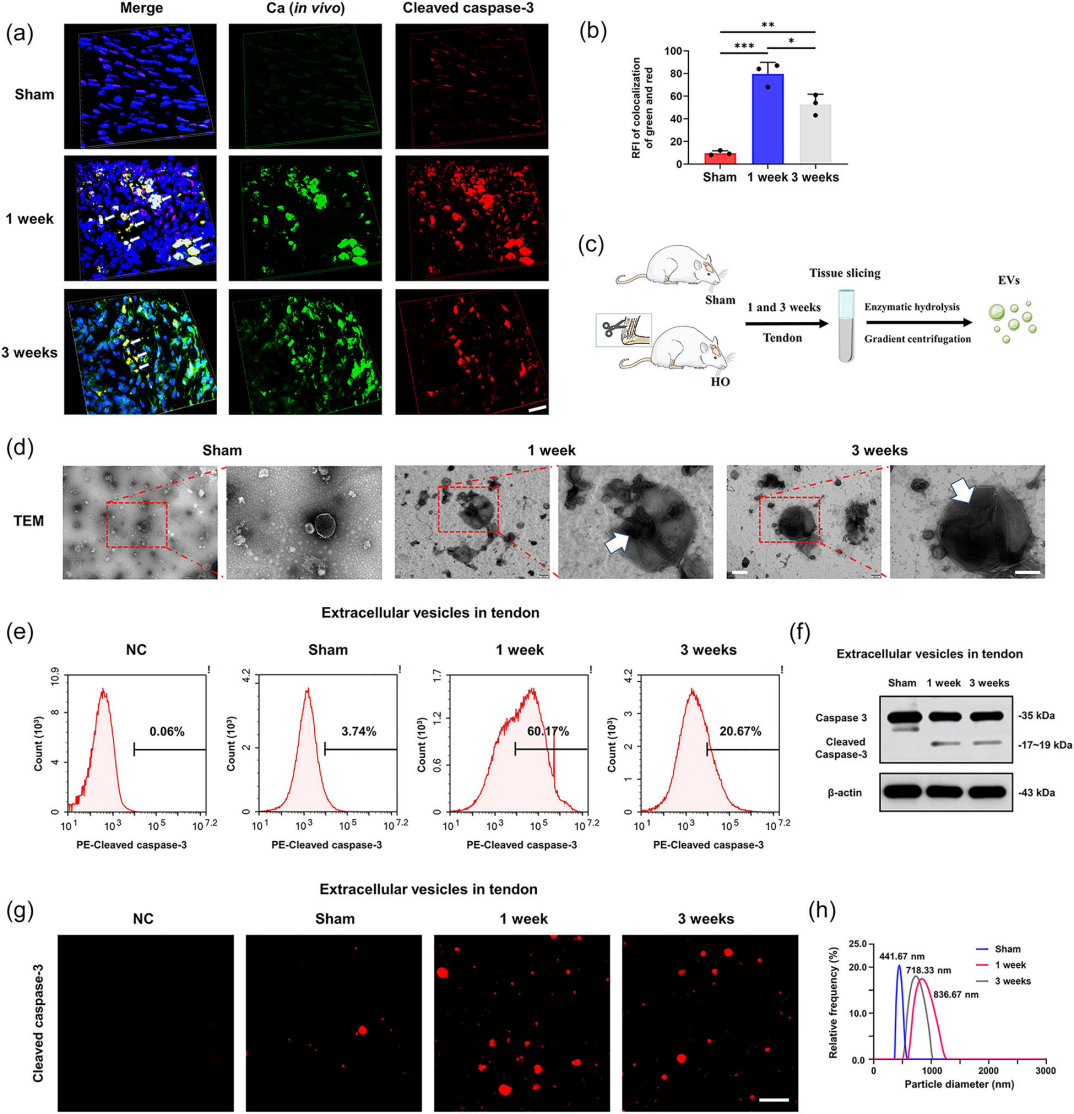
Characterization and isolation of the extracellular vesicles in sham and HO tendons. (a) Immunofluorescence microscopy of Achilles tendons from the sham and HO groups after 1 and 3 weeks. Ca (in vivo), green; Cleaved caspase‐3, red; DAPI, blue. Calcified apoVs were showed (arrows). Scale bar: 50 µm. (b) Quantitative analysis of the colocalization of the free green dots and red dots of Achilles tendons from sham and HO groups after 1 and 3 weeks (*n* = 3). RFI: relative fluorescence intensity. (c) Schematic representation of the isolation of the EVs in sham and HO tendons. (d) TEM images of the EVs of Achilles tendons from the sham and HO groups after 1 and 3 weeks. Scale bar: 1 µm. High magnification of the red rectangle outline in the low magnification image showing the EVs (arrows). Scale bar: 200 nm. (e) Flow cytometric analysis of cleaved caspase‐3 in the EVs. (f) Western blotting analysis showing the presence of Caspase‐3/Cleaved caspase‐3 in the EVs. (g) Representative confocal microscopy images of cleaved caspase‐3 staining in the EVs. Scale bars, 10 µm. (h) Nanoparticle‐tracking analyses (NTA) of EVs of Achilles tendons of rats from the sham and HO groups after 1 and 3 weeks (*n* = 3). Data are presented as the means ± standard deviations (*n* = 3). Statistical analyses were performed using one‐way ANOVA with a post‐hoc Tukey's test. *p < 0.05, **p < 0.01, ***p < 0.001.

To further explore the pathomechanism of tendon calcification, ultrastructure analysis of sham and HO tendons was performed. Magnified TEM images of 1 week HO tendons showed that the fibroblast released the apoVs in the ECM (Figure 3g, red arrows). Elemental analysis indicated that the apoVs contained both calcium and phosphate, and were thus termed as calcified apoVs (Figure S11). In the 3‐week HO tendon, nuclei was fragmented into discrete chromatin bodies because of nuclear DNA degradation. SEM images also demonstrated that the calcified apoVs aggregated into larger calcified nodules and were distributed into the ECM in the 1‐ and 3‐week HO groups. No calcified apoVs were observed in any tendon samples from the sham group (Figure 3h). In the 1‐week HO group, elemental mapping analysis also confirmed that the calcified apoVs released from the fibroblasts, or around the disorganized collagen matrix, contained calcium, phosphorus and nitrogen (Figure 3i). The calcified apoVs were also identified using calcein and cleaved caspase‐3 staining at different time points and the quantity of the calcified apoVs peaked at 1 week after injury (Figure 4a,b). Next, we isolated the EVs from tendons of sham and HO rats using the enzymatic hydrolysis and gradient centrifugation protocol and characterized them (Figure 4c). Flow cytometric analysis, TEM and immunofluorescent staining were further used to clarify the apoVs among the total EVs from tendons of sham and HO rats. The TEM results showed that the EVs from the 1 and 3 weeks HO tendons were larger and had an electron‐dense internal structure (Figure 4d). The flow cytometric analysis of cleaved caspase‐3, a specific marker of apoVs, showed that the percentage of the apoVs was 60.17% (1 week HO group) and 20.67% (3 weeks HO group) among the total EVs (Figure 4e). The presence of cleaved caspase‐3 in the apoVs in the 1 and 3 weeks HO group was also confirmed by western blotting and IF staining (Figure 4f,g). Nanoparticle tracking analysis revealed that the diameter of the EVs was 718.33 nm at 1 week post‐injury and 836.67 nm at 3 week post‐injury (Figure 4h). Collectively, these results indicated the potential effect of PROCR^+^ fibroblast‐released calcified apoVs on the early stage of HO calcification.

**FIGURE 5:**
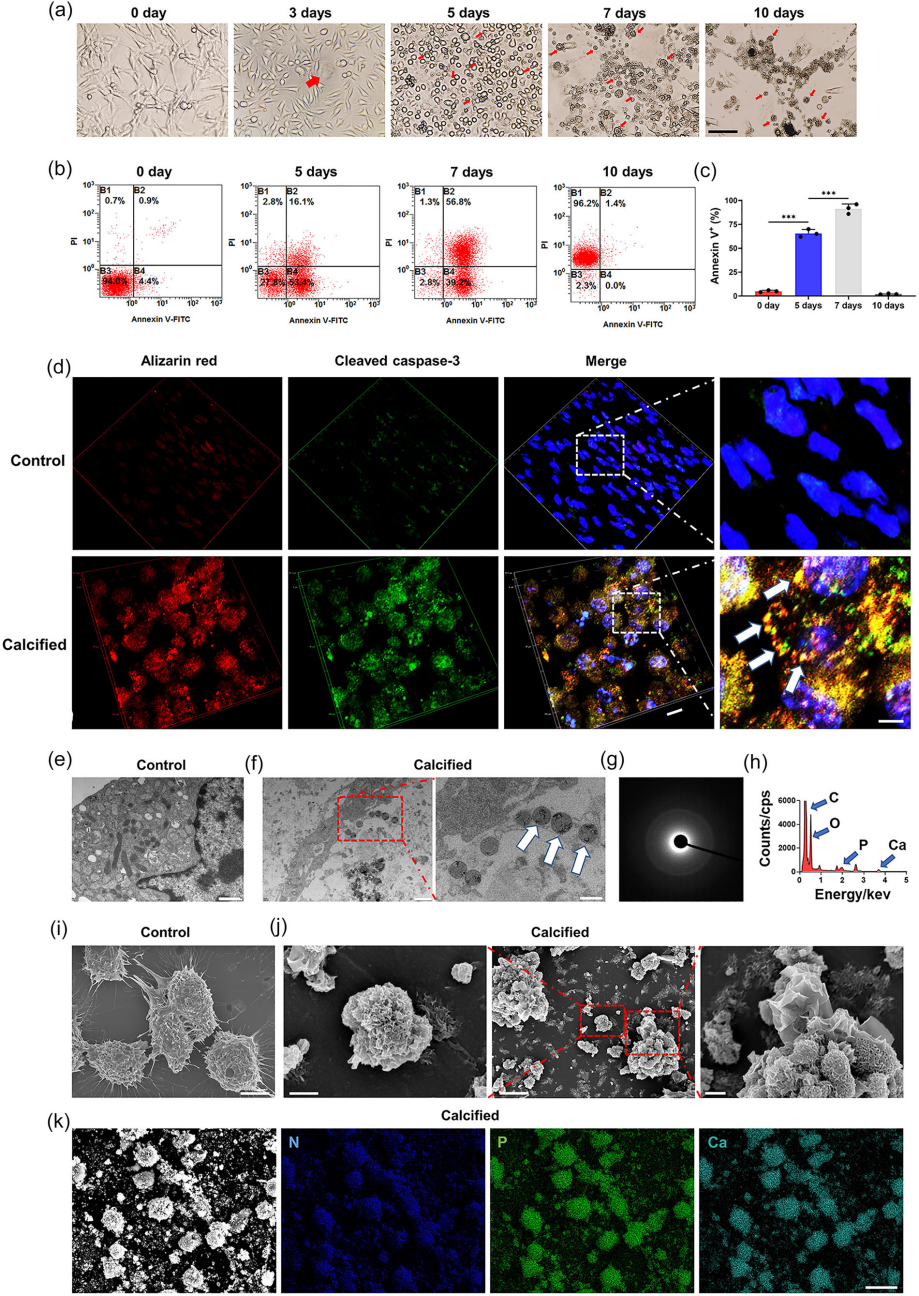
Local high calcium and phosphorus caused by the calcified apoVs aggravated its secretion from the fibroblasts. (a) Phase contrast microscope images of the fibroblasts cultured in the calcified medium for 0, 3, 5, 7 and 10 days. Scale bar, 100 µm. (b) Flow cytometry of Annexin V and PI of the fibroblasts cultured in the calcified medium for 0, 5, 7 and 10 days. (c) The corresponding quantification of the percentages of apoptotic fibroblasts cultured in the calcified medium for 0, 5, 7 and 10 days (*n* = 3). (d) Representative images of immunofluorescence staining of fibroblasts cultured in the control and calcified medium for 7 days. Alizarin red S, red; Cleaved caspase‐3, green; DAPI, blue. Scale bar, 10 µm. High magnification of the white rectangle outline in the low magnification image showing the calcified apoVs (arrows). Scale bar: 5 µm. (e) Representative TEM images of fibroblasts cultured in the control medium for 7 days. Scale bar: 1 µm. (f) Representative TEM images of fibroblasts cultured in the calcified medium for 7 days. Scale bar: 1 µm. High magnification of the red rectangle outline in the low magnification image showing the calcified apoVs (arrows). Scale bar: 500 nm. (g) Electron diffraction of the mineral precursors within the calcified apoVs. (h) Elemental analysis of the regions depicted by the arrows in (f). (i) Representative SEM images of fibroblasts cultured in the control medium for 7 days. Scale bar: 5 µm. (j) Representative SEM images of fibroblasts cultured in the calcified medium for 7 days. Scale bar: 3 µm. High magnification of the red rectangle outline in the low magnification image showing the calcified apoVs. Scale bar: 500 nm (left), 200 nm (right). (k) SEM images and elemental mapping showing the aggregation of calcified apoVs in the calcified group. Scale bar: 20 µm. Data are presented as the means ± standard deviations (*n* = 3). Statistical analyses were performed using one‐way ANOVA with a post‐hoc Tukey's test. ***p < 0.001.

Apoptotic fibroblast secretion of calcified apoVs resulted in large deposits of calcification in the ECM, which directly increased the stiffness of the microenvironment (Figure 3i). To further investigate the calcified apoVs, the fibroblasts were cultured in calcified medium with staurosporine, which showed significant morphological alteration (Figure 5a). Similar to what occurred in vivo in HO tendons (Figure 3g), small calcifications entrapped by the collagen matrix fused to form larger calcified nodules, and the calcified apoVs aggregated to produce spherical calcified structures (Figure 5a). Fibroblasts apoptosis was verified by the flow cytometric analysis, showing a time‐dependent manner and the proportion of apoptotic cells among the fibroblasts reached the peak at 7 days (96%) (Figure 5b,c). The calcified apoVs were also identified by alizarin red S and cleaved caspase‐3 co‐staining at 7 days (Figure 5d). TEM of fibroblasts cultured for 7 days showed that the calcified apoVs in the calcified group contained amorphous calcium and phosphorus (Figure 5e–h). In the calcified group, we observed the release of calcified apoVs from fibroblasts, which aggregated to produce calcified nodules with increased ECM stiffness (Figure 5i,j and Figures S12 and S13). Elemental mapping confirmed mineral Ca, P and N within the calcified apoVs (Figure 5k). These data suggested that the calcified apoV‐induced high calcium and phosphorus microenvironment in turn aggravated the secretion of calcified apoVs from fibroblasts, forming a positive feedback loop to promote the Achilles tendon calcification.

### 3.4 The calcified apoVs initiate calcification in vivo by enriching calcium via Annexin channels

**FIGURE 6:**
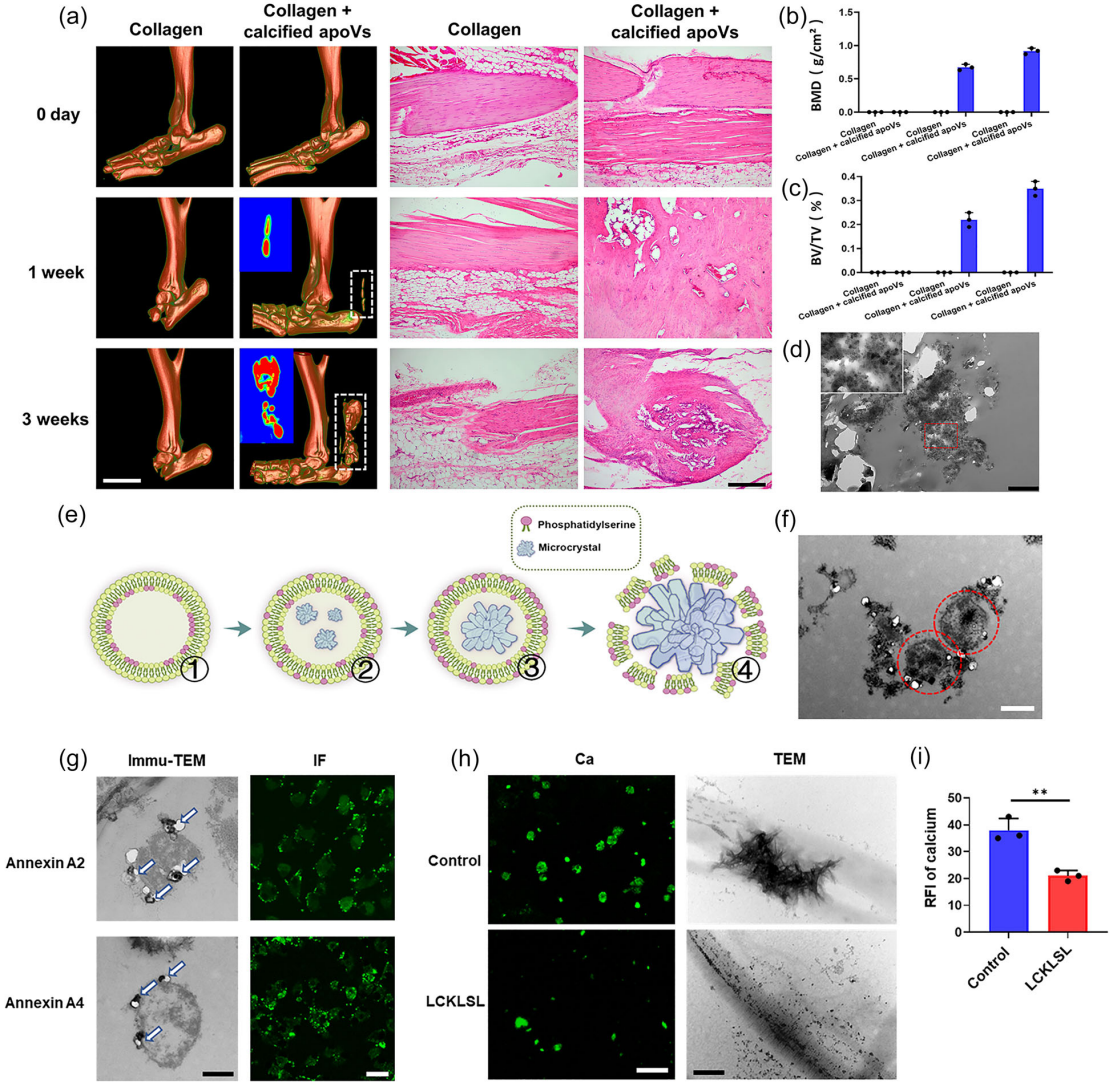
The calcified apoVs initiate calcification in vivo by enriching calcium through Annexin channels. (a) Micro‐computed tomography (micro‐CT) and H&E staining images of the rat intratendon implantation model from the collagen and collagen + calcified apoVs groups after 1 and 3 weeks. Scale bar, 5 mm (left); 100 µm (right). (b and c) Quantitative analysis of bone histomorphometric parameters based on the micro‐CT images in (a): (b) bone mineral density (BMD) and (c) bone volume/tissue volume (BV/TV). (d) TEM image of the mineral deposition within the calcified plaque in the collagen + calcified apoVs group (red rectangle). Scale bar: 1 µm. (e) Schematic representation of calcified apoVs‐mediated enrichment of calcium at the four stages. (f) Representative TEM image of calcified apoVs indicated with a red circle. Scale bar, 500 nm. (g) Immunogold and immunofluorescence labelling of Annexins on calcified apoVs (arrows) in the vicinity of the ECM calcification in the calcified group. Scale bar: 300 nm (left); 1 µm (right). (h) Fluorescence microscopy and TEM images of calcified apoVs incubated with collagen I for 3 days. Scale bar: 5 µm (left); 200 nm (right). (i) Quantitative analysis of the relative intensity of the fluorescence of calcium based on (h) (*n* = 3). Data are presented as the means ± standard deviations (*n* = 3). Statistical analyses were performed using Student's *t* test and two‐way ANOVA with post‐hoc Tukey's tests. ns, not significant. **p < 0.01.

To explore whether transplantation of calcified apoVs could directly initiate HO in vivo, the rat intratendon implantation model was used. The apoVs were isolated from fibroblasts and the characteristics and functions of the apoVs were examined. The isolated apoVs contained calcium and phosphorus (Figure S14A,B). The apoVs had a similar size to those seen in vivo in the HO tendons (Figure S14C). Flow cytometry analysis of cleaved caspase‐3, a specific marker of apoVs, showed that the percentage of the apoVs was 90.25% among the total EVs (Figure S14D). The expression of cleaved caspase‐3 in the apoVs was also verified by the confocal microscopy and western blotting (Figure S14E,F). Collagen scaffolds were then immersed in the calcified apoVs (1 mg/mL calcified apoVs, 3.5 mM calcium ions, 2.1 mM phosphate ions) and implanted into the intratendon pockets of rats. Similar sized collagen scaffolds that were immersed in the solution (3.5 mM calcium ions, 2.1 mM phosphate ions) were used as controls. After 1 week, Micro‐CT and 3D reconstruction of the dissected legs in the collagen + calcified apoVs group showed calcification of the collagen scaffolds, but not in the controls (Figure 6a). Ectopic bone was formed in the collagen + calcified apoVs group after 3 weeks (Figure 6a). Using H&E staining, we observed ectopic bone formation in rat intratendon implantation models. No ectopic bone was observed in the control group up to 3 weeks. A developed cancellous bone at 3 weeks post‐surgery was clearly identified in the collagen + calcified apoVs group, but not in the control group (Figure 6a). In the collagen + calcified apoVs group, the BV/TV ratios and BMD were significantly higher than those in the control group (*p* < 0.05, Figure 6b,c). TEM images and the results of element mapping and selected area electron diffraction indicated the mineral masses in the collagen scaffolds of the calcified apoVs group (Figure 6d and Figure S15). Collectively, these data indicated that the calcified apoVs contributed to ectopic calcification.

A calcification model was further developed using 3D self‐assembled collagen fibrils to understand the mechanism of calcified apoV‐induced calcification. The collagen fibrils were examined using TEM to monitor the evolution of calcified apoV‐mediated calcification in their native state. Mineral depositions were seen along the collagen fibrils as early as 3 days after immersion in calcification medium (Figure S16A). This provided compelling evidence that the calcified apoVs were adsorbed on the collagen fibrils and facilitated amorphous calcium phosphate (ACP) infiltration into the collagen fibrils, resulting in rapid calcification. Confocal laser scanning microscopy (CLSM) showed that the calcified apoVs were adsorbed on the collagen fibrils (Figure S16B), suggesting that calcified apoV‐collagen interaction might exist during the calcification. To investigate the interactions involved in calcified apoV‐collagen binding, we employed Molecular dynamic (MD) simulation to provide detailed microscopic modelling at the molecular scale. MD simulation confirmed that calcified apoVs binded to collagen fibrils (Figure S17A–F). Calculation of the binding energy between calcified apoVs and collagen attributed the energy to electrostatic interaction. The contact area between the calcified apoVs and collagen was 7.38 nm^2^, and the average electrostatic interaction energy, van der Waals interaction energy and total interaction energy were −3928.33, −184.93 and −4113.26 kJ/mol, respectively. Thus, electrostatic interaction is the predominant contributor to the calcified apoV‐collagen interaction at the overlap zone, and Arg11 and Arg14 of the 3 collagen chains contribute greatly to binding, whereas Glu13 repels proximity to the phospholipid membrane (Figure S17D–F).

A novel model system of culturing fibroblasts with high calcium and phosphorus medium was established to investigate the calcium uptake and release in calcified apoV‐mediated calcification. After 6 h, phosphatidylserine (PS, apoptotic marker) and Fluo‐4 (calcium marker) were clearly detectable, and were significantly upregulated after 12 h in the calcified group. The calcified apoVs were identified four stages: PS ectropion, calcium influx, crystal nucleon formation and membrane structure penetration. The high Ca concentration in the calcified apoVs triggers their precipitation. When the membrane structure of the calcified apoVs was ruptured, the calcification was released into the ECM (Figure 6e,f and Figure S18). The SEM results revealed that mineral deposition and hydroxyapatite crystallization evolve from intermediate amorphous or poorly crystalline phases inside the membrane structure to more crystalline apatite phases that penetrate the membrane (Figures S19 and S20). To explore the function of Ca enrichment in calcified apoVs, mass spectroscopy (MS) was used to identify calcified apoVs membrane proteins (Li, Xiao, et al., 2022). The MS/MS spectrum showed a series of ions in the mass range of 100–1800 that were derived from fragmentation of the peptide backbone. Proteomic analysis demonstrated that the calcified apoVs contained several ion channels. Ca^2^ **^+^** influx into calcified apoVs occurred through Annexin channels (Figures S21 and S22). Immunogold and immunofluorescence labelling of Annexins on calcified apoVs confirmed the presence of Annexin channels (Figure 6g). Importantly, blocking the Annexin channels using LCKLSL effectively inhibited the affinity of calcified apoVs for Ca^2+^ and their calcification (Figure 6h,i). Therefore, the calcified apoVs continuously enriched for calcium through Annexin channels, subsequently inducing calcification of the ECM.

### 3.5 The calcified apoV‐induced calcification in the early stage of HO promotes M2 macrophage polarization

**FIGURE 7:**
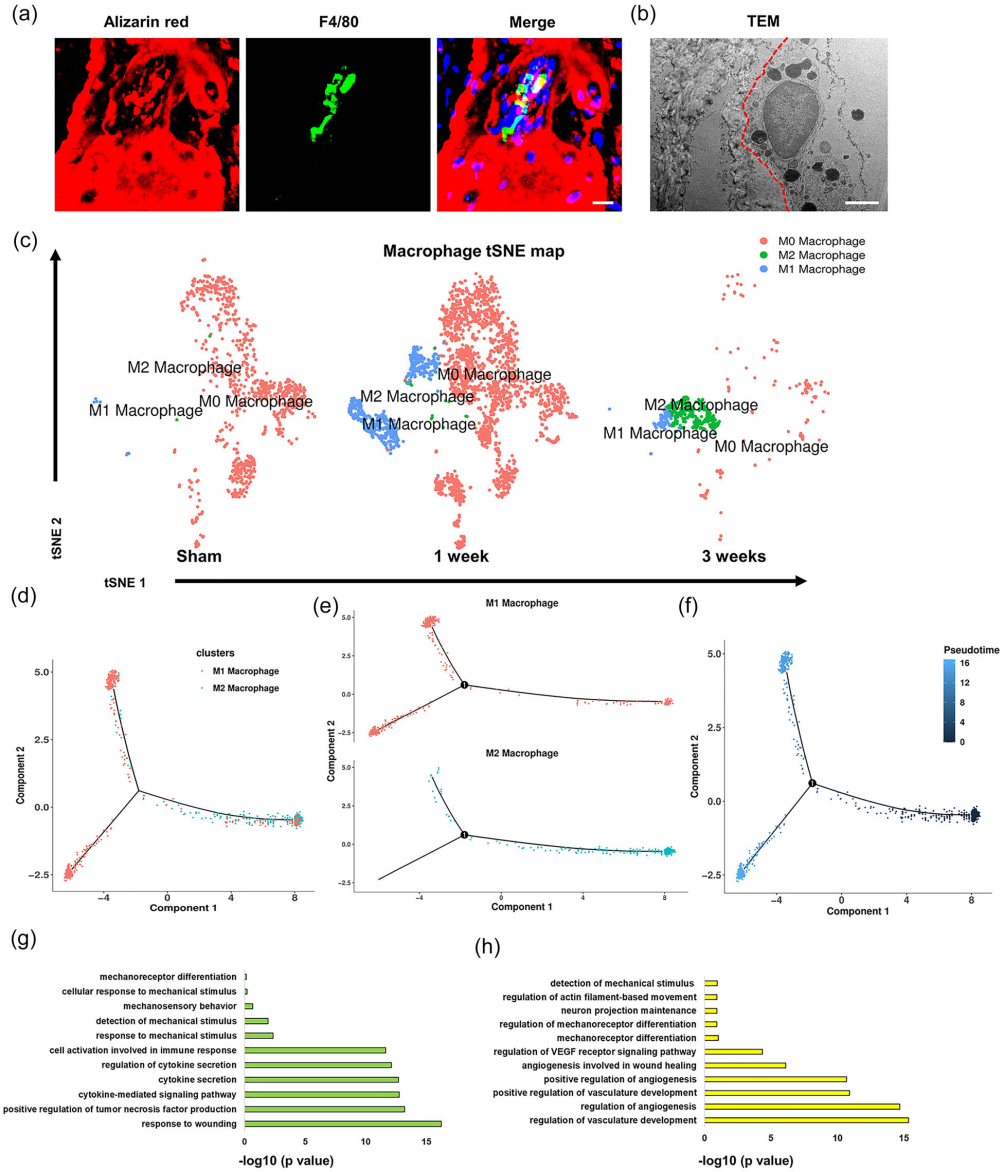
The calcification induced by the calcified apoVs in the early stage of HO promotes M2 macrophage polarization. (a) Representative confocal images of Achilles tendons stained with Alizarin red (red), F4‐80 (green) and DAPI (blue) in rat tendons in 1 week HO group. Scale bars, 30 µm. (b) Representative TEM images of Achilles tendons at 1 week HO group. The red dashed line indicates the contact interface between macrophages and the calcification. Scale bars, 1 µm. (c) tSNE of macrophage clusters (M0–M2 macrophage). Clusters were generated using a resolution of 0.2 before subclustering into major cell types according to the Methods. (d–f) Pseudotime analysis of macrophage clusters (M0–M2 macrophage). (g) Representative analysis of GO categories showing the different functions of M1 macrophage. (h) Representative analysis of GO categories showing the different functions of M2 macrophage.

Macrophage polarization promoted the progression of HO (Lucas et al., 2010; Wu et al., 2020; Zhang et al., 2020). Do the calcified apoVs released during HO initiation further affect macrophage polarization? Immunofluorescence staining and TEM showed that macrophages were located near the calcification of loose connective tissue at 1 week after Achilles tendon injury (Figure 7a,b). In our single cell data, analysis of monocyte/macrophage subpopulations identified three different clusters (Figure 7c). Heatmap analysis of gene expression in macrophage subclusters and subsequent pseudotime analysis revealed that the M1 cluster, situated at the earliest pseudotime, had the strongest proinflammatory capacity and later polarized into the M2 cluster with anti‐inflammatory lineage fates (Figure 7d–f and Figure S23). GO analyses showed that M1 macrophages associated with mechanical induction and inflammation, while M2 macrophages were associated with neurogenesis, angiogenesis and osteogenesis (Figure 7g,h).

To determine the potential role of stiffness in macrophage regulation in HO formation in injured Achilles tendons, we fabricated two kinds of polydimethylsiloxane (PDMS) substrates with different Young's moduli (stiff, 15:1, and soft, 45:1 (PDMS elastomer:curing agent)). We seeded bone marrow‐derived macrophages (BMDMs) onto the PDMS substrates to explore changes in cell behaviour (Figure S24). We simulated the inflammatory environment using inflammatory factors and detected M1 macrophage activation. Following lipopolysaccharide treatment, M1 phenotype macrophages were seeded onto the PDMS substrates with different stiffness. After cell attachment for 24 h, the macrophages showed a larger spreading area in the stiff group than in the soft group. In addition, the mRNA levels of *inos* and *Cd206* (M1 and M2 macrophage markers, respectively) were also detected, indicating that macrophages were activated and polarized from the M1 to the M2 phenotype in the stiff group. Furthermore, we detected relatively strong expression of angiogenesis and neurogenesis‐related genes in the stiff group (Figure S25). Thus, these results suggested that macrophages are a heterogeneous cluster with distinct pseudotime lineage fates, with either proinflammatory or anti‐inflammatory phenotypes. The calcified apoV‐induced calcification promoted the polarization of M1 to M2 macrophages. To explore the potential role of calcified apoVs in macrophage regulation, we co‐cultured DiR‐labelled calcified apoVs and phalloidine‐labelled M1 macrophages onto the soft and stiff PDMS substrates, and the protein and mRNA levels of Cd206 (M2 macrophage marker) in macrophages were then detected (Figure S26). Immunofluorescent staining identified that the calcified apoVs were phagocytized by the macrophages in both soft and stiff substrates groups, and the negative control (NC) group verified that there was no free dye contamination (Figure S26). Notably, in the soft substrate group the phagocytosis of calcified apoVs did not change the levels of Cd206 in macrophages, while in the stiff substrate group the protein and mRNA levels of Cd206 in macrophages were both significantly increased (Figure S26). These results demonstrated that the stiff substrate was necessary in the M1 to M2 polarization of macrophages in response to calcified apoVs. Therefore, the calcified apoVs in the initiation stage and macrophages in the progression stage represent therapeutic targets for HO.

### 3.6 Inhibition of calcified apoV release and macrophage depletion delay the initiation and progression of HO, respectively

**FIGURE 8:**
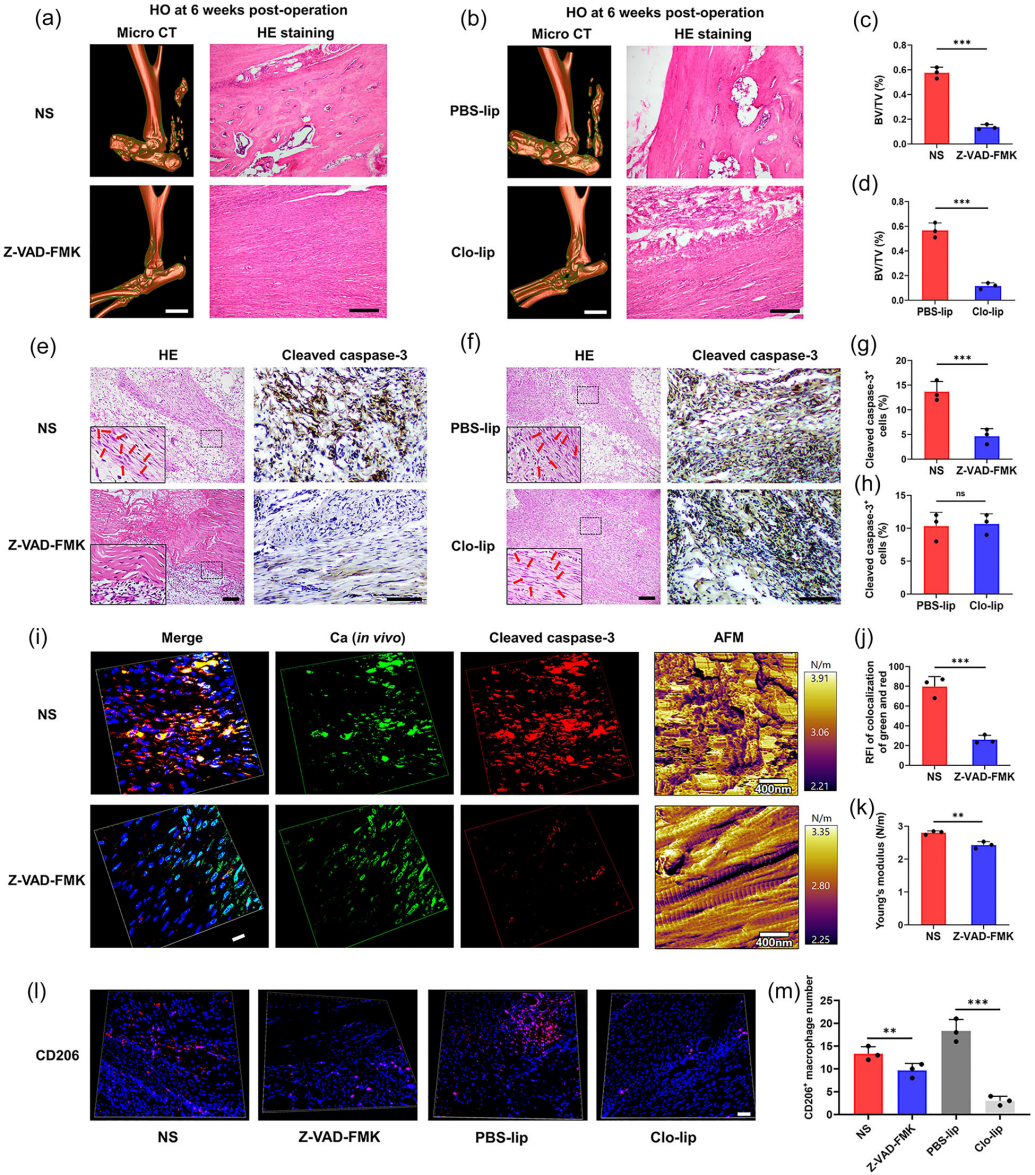
Inhibition of calcified apoV release and macrophage depletion delay the initiation and progression of HO, respectively. (a and b) Micro‐CT images (scale bars, 5 mm) and H&E staining (scale bars, 100 µm) of heterotopic calcification in the Achilles tendons 6 weeks after surgery from the NS, z‐VAD‐fmk, PBS‐lip and Clo‐lip groups. (c and d) Quantification of the BV/TV based on (a and b). (e and f) Representative images of H&E staining and immunohistochemical staining of the Achilles tendons derived from the NS, z‐VAD‐fmk, PBS‐lip and Clo‐lip groups. Scale bar, 50 µm. (g and h) Quantitative analysis of cleaved caspase‐3^+^ cells in (e and f) (*n* = 3). (i) Immunofluorescence microscopy and representative AFM stiffness map of Achilles tendons of rats from the NS and z‐VAD‐fmk groups. Ca, green; Cleaved caspase‐3, red; DAPI, blue. Scale bar: 10 µm. (j) Quantitative analysis of the colocalization of the free green dots and red dots of Achilles tendons of rats (*n* = 3). RFI: relative fluorescence intensity. (k) Quantitative analysis of AFM stiffness based on (i) (*n* = 3). (l) Representative confocal images of M2 macrophages (CD206 positive) of Achilles tendons of the rats from the NS, z‐VAD‐fmk, PBS‐lip and Clo‐lip groups. Scale bars, 50 µm. (m) Quantitative analysis of macrophage phenotype from the NS, z‐VAD‐fmk, PBS‐lip and Clo‐lip groups. Data are presented as the means ± standard deviations (*n* = 3). Statistical analyses were performed using Student's *t* test and one‐way ANOVA with a post‐hoc Tukey's test. ns, no significance. *p < 0.05, **p < 0.01, ***p < 0.001.

Next, we investigated whether inhibition of calcified apoV release and macrophage depletion were potential therapeutic targets for HO. Caspases play an essential role in apoptosis; therefore, their inhibition with the caspase inhibitor z‐VAD‐fmk has been used to reduce apoptotic cell death. Micro‐CT and H&E staining were used to examine Achilles tendons harvested from rats at 6 weeks after achillotenotomy. In addition to lamellar bone, bone marrow and trabecular bones were also seen in the formed ossifications. In contrast, the z‐VAD‐fmk treated rats failed to induce ectopic mineralization after apoptosis inhibition and quantitative analysis of BV/TV also identified a significant difference (Figure 8a,c). H&E staining showed that at 1 week post‐surgery, apoptosis of fibroblasts was partially inhibited by z‐VAD‐fmk within the Achilles tendon. In addition, the fibroblasts underwent necrosis after apoptosis inhibition (Figure 8e). Cleaved caspase‐3 was clearly detectable in normal saline (NS) group but was significantly downregulated in z‐VAD‐fmk group (Figure 8e,g). To determine the distribution of calcified apoVs during HO formation in the z‐VAD‐fmk group, immunofluorescent staining of the sections of injured tendon tissues from HO rats was performed. At 1 week post‐surgery, the calcified apoVs were induced as expected in the NS group, whereas significantly decreased calcified apoVs levels were observed in the z‐VAD‐fmk group (Figure 8i,j). AFM revealed partial inhibition of the calcification associated with increased stiffness by z‐VAD‐fmk (Figure 8i,k). Z‐VAD‐fmk also inhibited the polarization of M2 macrophages (Figure 8l,m). These results suggested that apoptosis was required for calcification in the early stage of injury and the later stage of bone formation.

To verify the importance of macrophages to HO formation in injured Achilles tendons, rats that underwent Achilles tenotomy were injected intravenously with clodronate–liposomes to deplete macrophages, using phosphate‐buffered saline (PBS)‐liposomes as controls. Micro‐CT showed that the clodronate‐liposomes significantly reduced the HO volume compared with that in the PBS‐liposomes group at 6 weeks post‐surgery (Figure 8b). The BV/TV in the clodronate–liposomes group was markedly lower than that in the PBS‐liposomes group during HO pathogenesis (Figure 8b,d). Similarly, H&E staining showed a decreased in mature bone tissues in the clodronate–liposomes group compared with that in the PBS‐liposomes group in the injured Achilles tendons (Figure 8b). Fibroblast apoptosis and cleaved caspase‐3 levels were unaffected in the clodronate–liposomes group, while M2 macrophages were significantly depleted (Figure 8f,h,l,m). Collectively, these results suggested that inhibition of calcified apoVs release and macrophage depletion delayed the initiation and progression of HO, respectively.

## 4 DISCUSSION

In the present study, we for the first time identified that calcified apoVs, specifically secreted by PROCR^+^ fibroblasts in early stage of HO, led to tendon ECM calcification and stiffening and initiated the osteogenic cascade in HO. Specifically, calcified apoVs enriched calcium by annexin channels, absorbed to collagen I via electrostatic interaction, and aggregated to produce calcifying nodules in the tendon ECM leading to the calcification and stiffening of ECM. More importantly, apoV‐releasing inhibition or macrophage deletion both successfully reversed the HO. Our results identified the novel calcifying function and process of apoVs, revealed their pathogenic effects on HO development, and proposed a good target for early intervention in ectopic mineralization diseases.

It has been reported that the apoptotic bodies derived from vascular smooth muscle cell could act as nucleating structures for calcium crystal formation (Proudfoot et al., 2000), and those from sodium nitroprusside‐treated chondrocytes could precipitate calcium from solution (Hwang & Kim, 2015). While those previous studies focused on the apoptotic bodies (1–5 µm in diameter) in the mineralization process, an increasing number of studies have identified smaller membrane‐bound vesicles secreted during apoptosis, termed as apoVs (<1 µm in diameter) (Azoidis et al., 2018; Bommanavar et al., 2020; Hasegawa et al., 2017; Li, Wu, et al., 2022). These apoVs have been revealed to have unique biological and functional characteristics that are emerging as crucial regulators for diverse biological processes, but their role in calcification are unknown (Fu et al., 2023; Ma et al., 2022; Ou et al., 2022; Qu et al., 2022; Wang et al., 2022; Zhang et al., 2022). Here, we for the first time demonstrated the calcifying function of apoVs (calcified apoVs) which played an important role in the formation of tendon calcification in the early stage of HO. Generally, apoVs are considered to modulate the biological functions of neighbouring cells by releasing a variety of substances, including lipids and proteins, that initiate signal transduction (Fu et al., 2023). Therefore, the identified calcified apoVs extends the current understanding of apoVs and sheds light on the prevention and treatment of calcification related diseases. More importantly, we further found that those calcified apoVs mainly derived from apoptosis‐associated PROCR^+^ fibroblasts. It is very hard to intervene in the early stage of HO by targeting calcified apoVs, while our results provide a novel therapeutic strategy for early HO blockage by targeting the apoptosis and/or apoV secretion of PROCR^+^ fibroblasts. Further studies thus are needed to explore the detail mechanism of apoptotic signalling and apoVs secretion of PROCR^+^ fibroblasts for accurate therapy of early HO.

In the present study, we isolated the EVs from the tendons using the enzymatic hydrolysis and gradient centrifugation protocol (Crescitelli et al., 2021), and the flow cytometric analysis of cleaved caspase‐3 showed that the percentage of the apoVs was 60.17% among the total EVs in 1 week HO group (Figure 4e). More importantly, the apoVs tendon injection experiment has confirmed that the apoVs induced the calcification (Figure 6a), which verified that the apoVs in the early stage of HO initiate the calcification. And we will explore to separate the apoVs from the tendons in the future study. In previous studies, the apoVs were collected from the cell supernatant after the induction of apoptosis, and the vesicles secreted during the induction of apoptosis are considered as the apoVs for further study (Fu et al., 2023; Ma et al., 2022; Zheng et al., 2021). Although we did not strictly prove that the apoVs were released by apoptotic cells, we will further investigate the apoVs by labelling the apoVs in the apoptotic cells in the future study. Because the detecting size distribution of flow nanoanalyzer is between 20–200 nm and the diameter of the calcified apoVs was 700–900 nm, the conventional flow cytometry is more suitable for the detection of calcified apoVs rather than the flow nanoanalyzer. Actually, previous studies have successfully used the conventional flow cytometry to study the function of MSC‐derived apoVs (Zhang et al., 2022). Therefore, the conventional flow cytometry was used here to detect calcified apoVs.

Although the pathological changes to the microenvironment underlying HO are complex, our group has focused on the formation of calcification in early stage of injury and the stiffness of the microenvironment (Convente et al., 2018; Kraft et al., 2016). In atherosclerosis, the formation of calcifications causes plaque instability because of the high stress accumulation within the cap matrix, favouring cavitation events that occur owing to the large modulus mismatch between the stiff calcifications and the surrounding collagen (Hutcheson et al., 2016). In our study, multiple micro‐nano analytical technologies were applied to study the process of calcified apoVs induced calcification. The calcium deposition started with spherical mineral particle formation in the calcified apoVs, followed by densely packed material transformation deep into the ECM tendon. In the early stage of Achilles tendon injury, a large amount of local cell death occurs, releasing a large amount of calcium and phosphorus ions into the ECM, and our data also showed a large increase in calcium and phosphorus concentrations in the early stage of Achilles tendon injury, which was confirmed using in vitro experiments. The calcification in the injured tendon largely increased the stiffness of ECM around local cells, and the increased stiffness triggered mesenchaymal stem cells (MSCs) osteogenic differentiation by Hippo signalling pathway (Li et al., 2021), and promoted the formation of a local microenvironment favouring neurogenesis and angiogenesis, resulting the heterotopic calcification. Therefore, the abnormal outcome of cell fate and the consequent change to the microenvironment lead to the occurrence of HO.

Previous studies have demonstrated that macrophages regulate the critical processes of HO development, including osteogenic differentiation and chondrogenic differentiation of MSCs, hypoxic microenvironment and angiogenesis (Barruet et al., 2018; Eisenstein et al., 2018; Feng et al., 2020; Genêt et al., 2015; Huang et al., 2021; Kan et al., 2019; Wang et al., 2018; Zhang et al., 2021). Our results showed that depletion of macrophages greatly suppressed the development of HO, which was in accordance with previous studies (Wang et al., 2018). However, macrophage deletion could also have a substantial impact on the microenvironment. Given the high heterogeneity and plasticity of macrophages, the inhibitory effects of different phenotypes of macrophages on bone formation and osteoinhibition vary across studies (Tirone et al., 2019). For example, in the injured skeletal muscle, macrophage depletion shifts the fate of endothelial cells toward endochondral differentiation, and leads to ectopic bone formation (Tirone et al., 2019). Therefore, the mechanisms of ectopic bone formation promoted by macrophages are diverse and complex, and it is important to explore the full landscape of molecular and cellular mechanisms by which macrophages induce HO. To clarify whether macrophage polarization occurs after the phagocytosis of calcified apoVs or in response to the changes of the ECM stiffness, we co‐culturedDiR‐labelled calcified apoVs and phalloidine‐labelled M1 macrophages onto the soft and stiff PDMS substrates. The results showed that the phagocytosis of calcified apoVs did not cause macrophage M2 polarization, while the stiff substrate activated the macrophages polarized from the M1 to the M2 phenotype. It has been reported that during liver macrophage homeostasis apoV engulfment induce the polarization of macrophages towards the M2 phenotype, and the enriched proteins in apoVs such as cAMP‐dependent protein kinase type II‐alpha regulatory subunit (PRKAR2A) and receptor of activated protein C kinase 1 (RACK1) provides a molecular basis for the biological effects of apoVs (Zheng et al., 2021). Because apoVs are enriched with a set of functional proteins that are highly related to cellular behaviours and signal transduction, their function are highly heterogeneous depends on the specific microenvironment (Fu et al., 2023; Ma et al., 2022; Wang et al., 2022). Our findings extend the current understanding of apoVs function showing that the calcified apoVs from the early stage of HO could directly calcify and stiffen the tendon ECM, resulting in the M2 polarization of macrophages and thereafter osteogenic cascade in the HO microenvironment.

## 5 CONCLUSION

No strategies or drugs are available to prevent crystal deposition and permit mineral dissolution in HO tendons, emphasizing the importance of revealing the pathogenic mechanism of the HO microenvironment to identify therapeutic targets. This study showed that in the early stage of tendon injury, calcified apoVs from PROCR^+^ fibroblasts induced calcification and stiffening of the Achilles tendon, promoting M2 macrophage polarization and further initiated the osteogenic cascade during HO progression. A better understanding of these phenomena could result in new strategies to control the calcification process by inhibiting the early stages of hydroxyapatite deposition induced by calcified apoVs.

## AUTHOR CONTRIBUTIONS

**Jianfei Yan**: Formal analysis (equal); methodology (equal); visualization (equal); writing—original draft (equal). **Bo Gao**: Data curation (equal); methodology (equal); visualization (equal); writing—original draft (equal). **Chenyu Wang**: Formal analysis (equal); methodology (equal); visualization (equal); writing—original draft (equal). **Weicheng Lu**: Data curation (equal); formal analysis (equal); methodology (equal). **Wenpin Qin**: Data curation (equal); formal analysis (equal); methodology (equal). **Xiaoxiao Han**: Data curation (equal); formal analysis (equal); methodology (equal). **Yingying Liu**: Data curation (equal); formal analysis (equal); investigation (equal). **Tao Li**: Data curation (equal); formal analysis (equal); investigation (equal). **Zhenxing Guo**: Data curation (equal); formal analysis (equal); investigation (equal). **Tao Ye**: Data curation (equal); formal analysis (equal); investigation (equal). **Qianqian Wan**: Data curation (equal); formal analysis (equal); investigation (equal). **Haoqing Xu**: Data curation (equal); formal analysis (equal); investigation (equal). **Junjun Kang**: Investigation (equal); methodology (equal). **Naining Lu**: Investigation (equal); methodology (equal). **Changhe Gao**: Investigation (equal); methodology (equal). **Zixuan Qin**: Investigation (equal); methodology (equal). **Chi Yang**: Conceptualization (equal); supervision (equal); validation (equal); visualization (equal); writing—review and editing (equal). **Jisi Zheng**: Validation (equal); visualization (equal). **Pei Shen**: Validation (equal); visualization (equal). **Lina Niu**: Supervision (equal); validation (equal); visualization (equal); writing—review and editing (equal). **Weiguo Zou**: Supervision (equal); validation (equal); visualization (equal); writing—review and editing (equal). **Kai Jiao**: Funding acquisition (equal); supervision (equal); validation (equal); visualization (equal); writing—review and editing (equal).

## CONFLICT OF INTEREST STATEMENT

The authors declare no conflict of interest.

## Supporting information

Supporting Information

## Data Availability

All data needed to evaluate the conclusions in the paper are present in the paper and/or the Supplementary Information.
