# Supplementary material for: Calcified apoptotic vesicles from PROCR+ fibroblasts initiate heterotopic ossification
Source: J Extracell Vesicles. 2024 Apr 9;13(4):e12425. doi: 10.1002/jev2.12425 (PMC11004040; doi:10.1002/jev2.12425)
Supplement: Supplementary file 1 — Supporting Information [file JEV2-13-e12425-s001.docx]

**Calcified apoptotic vesicles from PROCR^+^ fibroblasts initiate heterotopic ossification**

**This PDF file includes:**

Fig. S1. Flow chart depicting the sequence of experiments conducted in the present study.

Fig. S2. SEM-EDS analysis of the element distribution of calcium (Ca), phosphorus (P), oxygen (O) and carbon (C) in the tendon from 1-week and 3-week HO groups.

Fig. S3. Schematic representation of the sham and HO rats injected with calcein.

Fig. S4. Micro-infrared analysis of sham and HO tendons in 1 and 3 weeks.

Fig. S5. AFM images of sham, 1 week and 3 week HO tendon showing Amplitude, ZSensor Retrace and Indentation Retrace modality.

Fig. S6. The 5 principal cell types in tendons have marked expression variations.

Fig. S7. Characterization of the PROCR^+^ fibroblast in the tendon.

Fig. S8. The histogram of the biological processes of different fibroblast clusters (GO).

Fig. S9. The histogram of the biological processes of different fibroblast clusters (KEGG).

Fig. S10. Characterization of the apoptosis of the PROCR^+^ fibroblast in sham and HO tendons.

Fig. S11. EDS elemental analysis of the selected area of the calcified apoVs containing electron-dense granules.

Fig. S12. Representative SEM and TEM images of the fibroblasts cultured in the calcified medium.

Fig. S13. AFM measurements of the fibroblasts cultured in the control and calcified medium for 7 days.

Fig. S14. The isolation and characterization of the apoVs.

Fig. S15. Element mapping and selected area electron diffraction of the collagen scaffolds of the calcified apoVs group.

Fig. S16. (A) TEM images of the calcified apoVs incubated with the collagen I for 3 and 5 days. (B) Immunofluorescence microscopy of the collagen I hydrogels after introduction of green-labeled calcified apoVs at 48 h.

Fig. S17. Molecular dynamic (MD) simulation analysis between the calcified apoVs and collagen I.

Fig. S18. Immunofluorescence microscope of the fibroblasts with high calcium and phosphorus concentrations medium for 6 and 12 hours.

Fig. S19. The calcified apoVs continuously enriched calcium and released the calcification.

Fig. S20. Time-lapse images monitoring the calcified apoVs enriching calcium and the localization of Fluo-4 staining in the calcified apoVs.

Fig. S21. The unique peptides of the top 5 scores in LC-MS/MS analysis of membrane protein of the calcified apoVs from the fibroblasts with high calcium and phosphorus concentrations medium.

Fig. S22. List of candidates with top 5 scores in LC-MS/MS analysis of membrane protein of the calcified apoVs from the tendon.

Fig. S23. The polarization of macrophage from M1 to M2.

Fig. S24. The macrophage cultured in two kinds of PDMS substrates with different young’s moduli.

Fig. S25. Increased stiffness of ECM promoted the polarization of macrophage from M1 to M2.

Fig. S26. The effect of the calcified apoVs on the polarization of macrophages to M2.

Table S1. Primers for qRT-PCR.

Supplementary figures

**
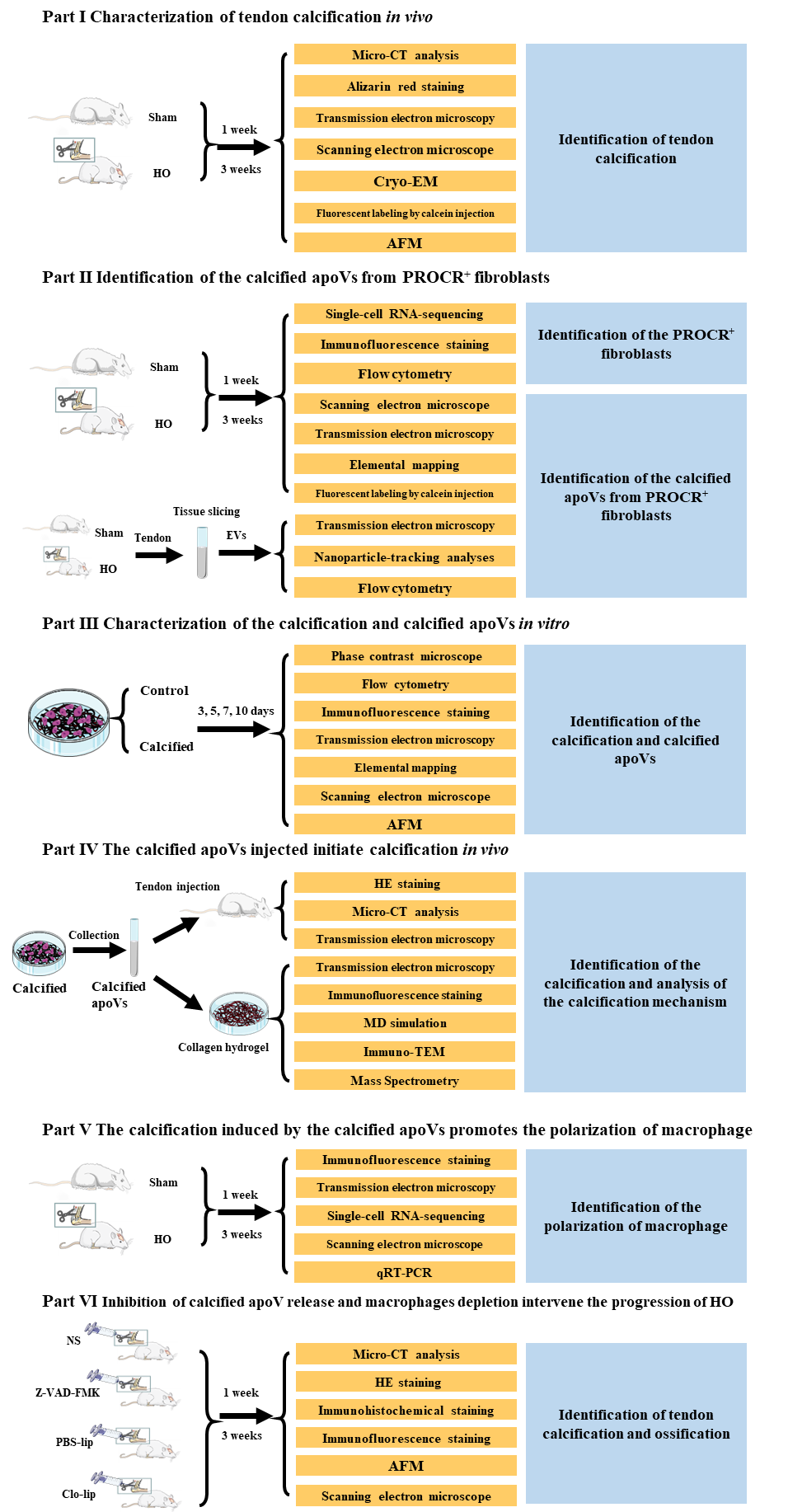
**

**Fig. S1**. Flow chart depicting the sequence of experiments conducted in the present study.


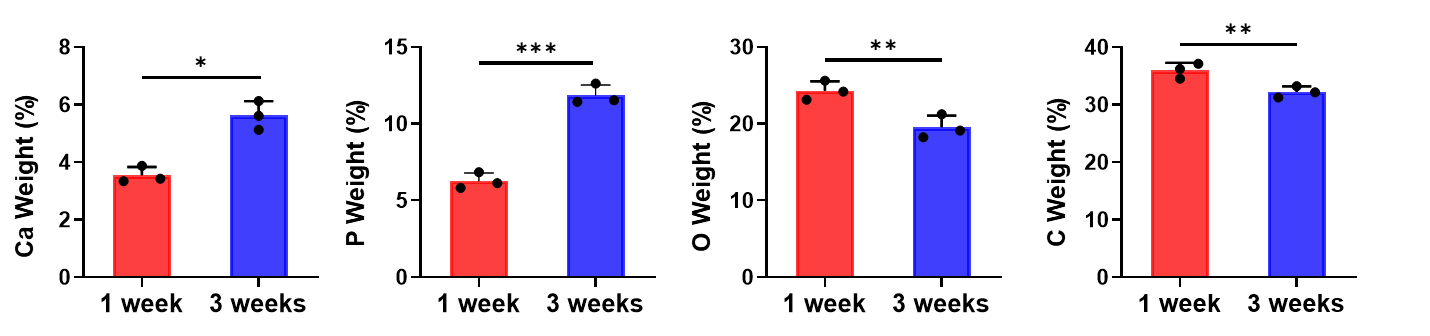


Fig. S2. SEM-EDS analysis of the element distribution of calcium (Ca), phosphorus (P), oxygen (O) and carbon (C) in the tendon from 1-week and 3-week HO groups (n = 3). All data was presented as mean ± standard deviation. Statistical analyses are performed by Student’s t test. *, *P* < 0.05; **, *P* < 0.01; ***, *P* < 0.001.


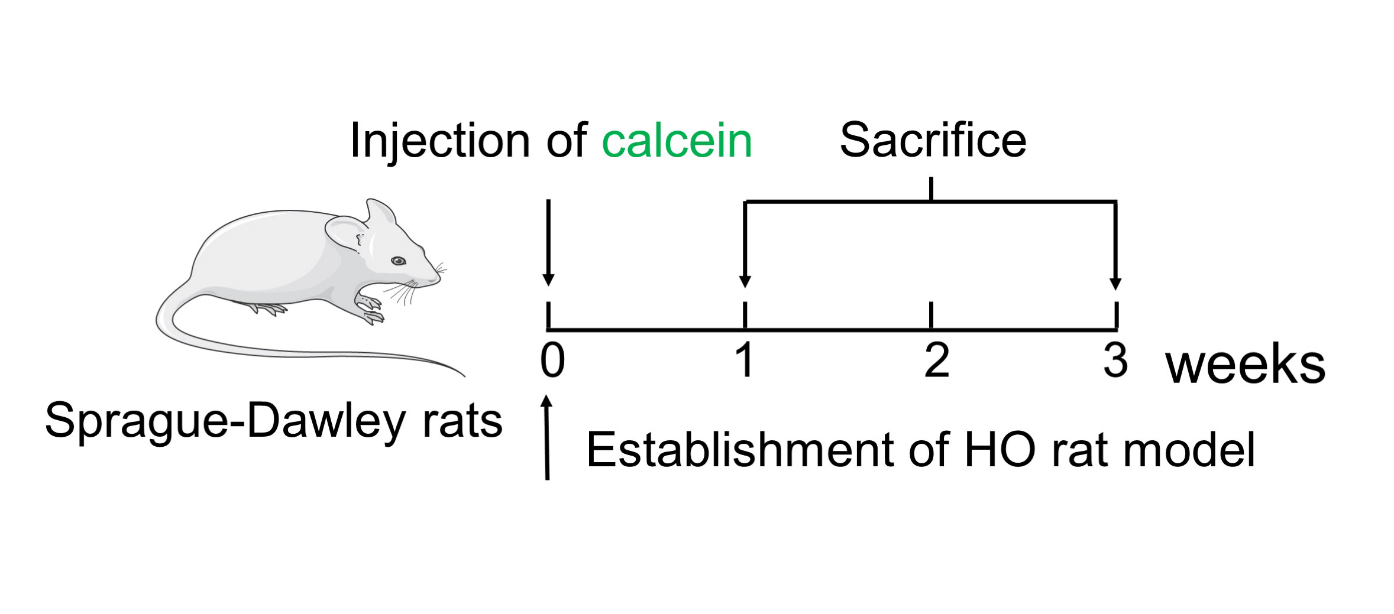


Fig. S3. Schematic representation of the sham and HO rats injected with calcein to record the formation of calcifications. Eight week old rats received achillotenotomy and an injection of calcein green. Injection of calcein green allowed tracing of the calcific mineral present at the initial time point. The rats were euthanized at 1 and 3 weeks after injection respectively.


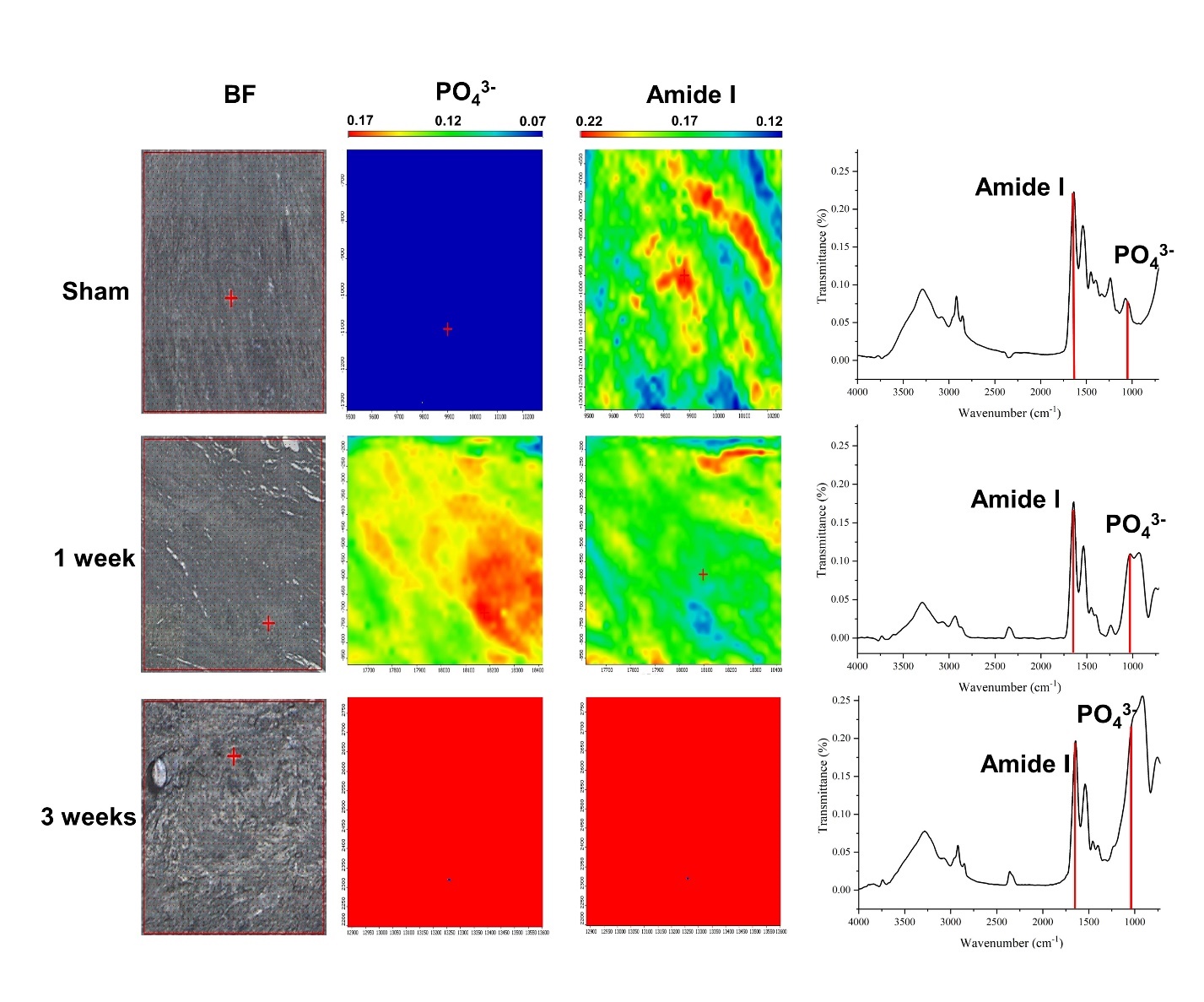


Fig. S4. Micro-infrared analysis of sham and HO tendons in 1 and 3 weeks. Infrared cartography and spectra of Amide I and PO_4_^3-^ in the sham, HO tendons (1 week and 3 weeks) were obtained.


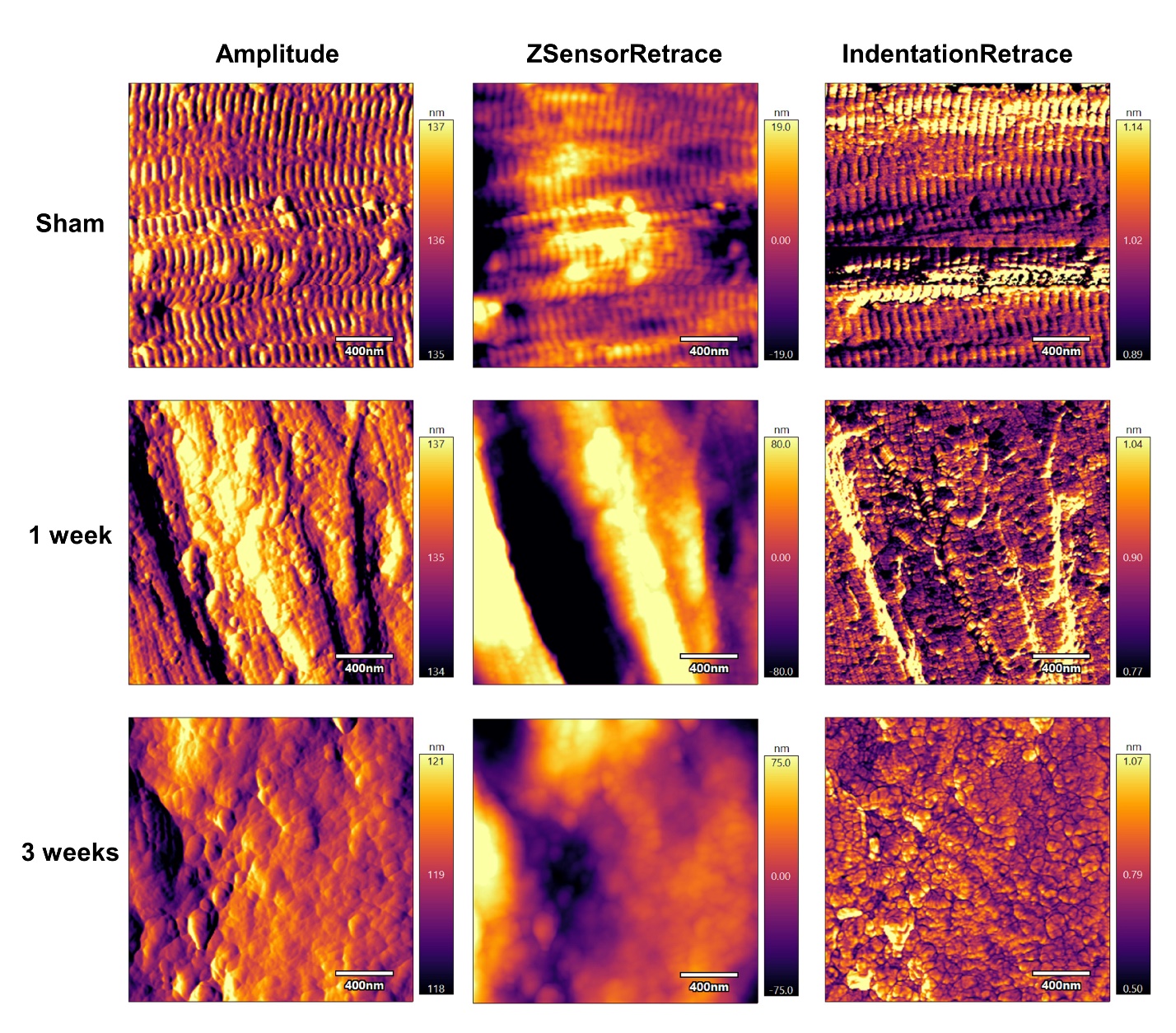
Fig. S5. AFM images of sham, HO (1 week and 3 weeks) tendon showing Amplitude, ZSensor Retrace and Indentation Retrace modality. AFM contact images were obtained in air using soft silicon nitride cantilevers with pyramidal tips. All images were processed using a first-order plane-fit function.


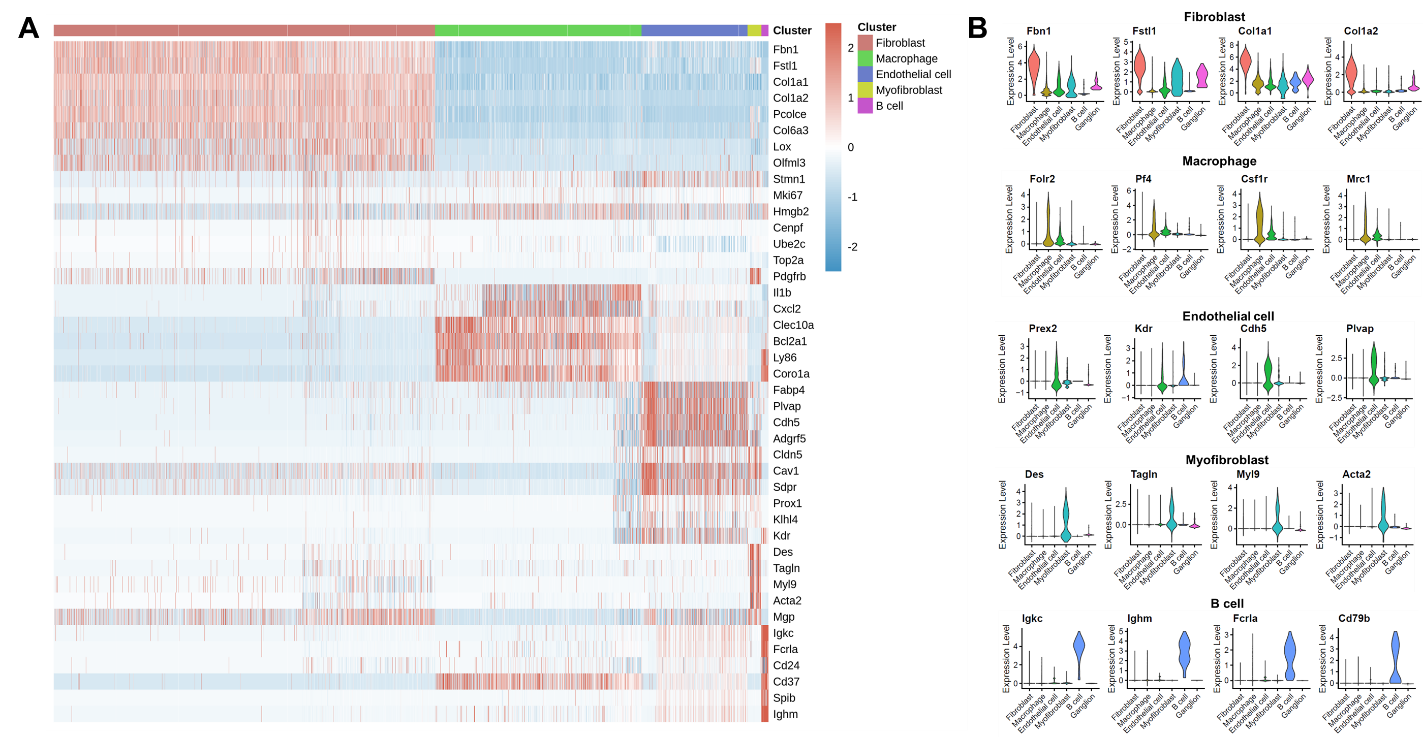


**Fig. S6**. The 5 principal cell types in tendons have marked expression variations. (**A**) Heatmap of the top 20 genes of the 5 main cell types in tendons. Expression values are normalized and scaled averages. (**B**) Violin plot of the expression of selected differentially expressed genes (DEGs) of fibroblasts, macrophages, endothelial cells, myofibroblasts and B cells.


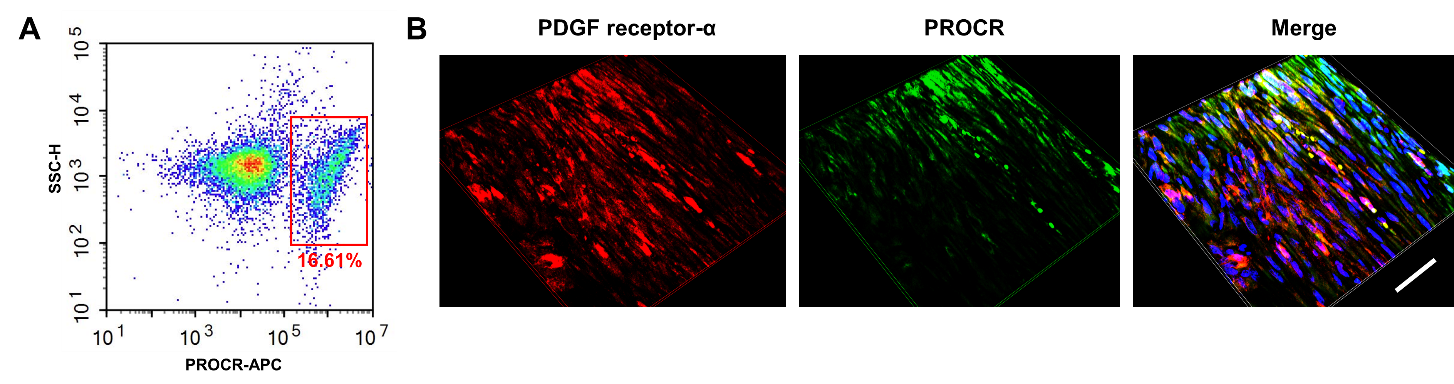


Fig. S7. Characterization of the PROCR^+^ fibroblast in the tendon. (A) FACS gating strategy of PROCR^+^ cells from Achilles tendons of the sham group. (B) Immunofluorescence microscopy of Achilles tendons of rats from the sham group. PDGF receptor-α, red; PROCR, green; DAPI, blue. Scale bar: 30 μm.


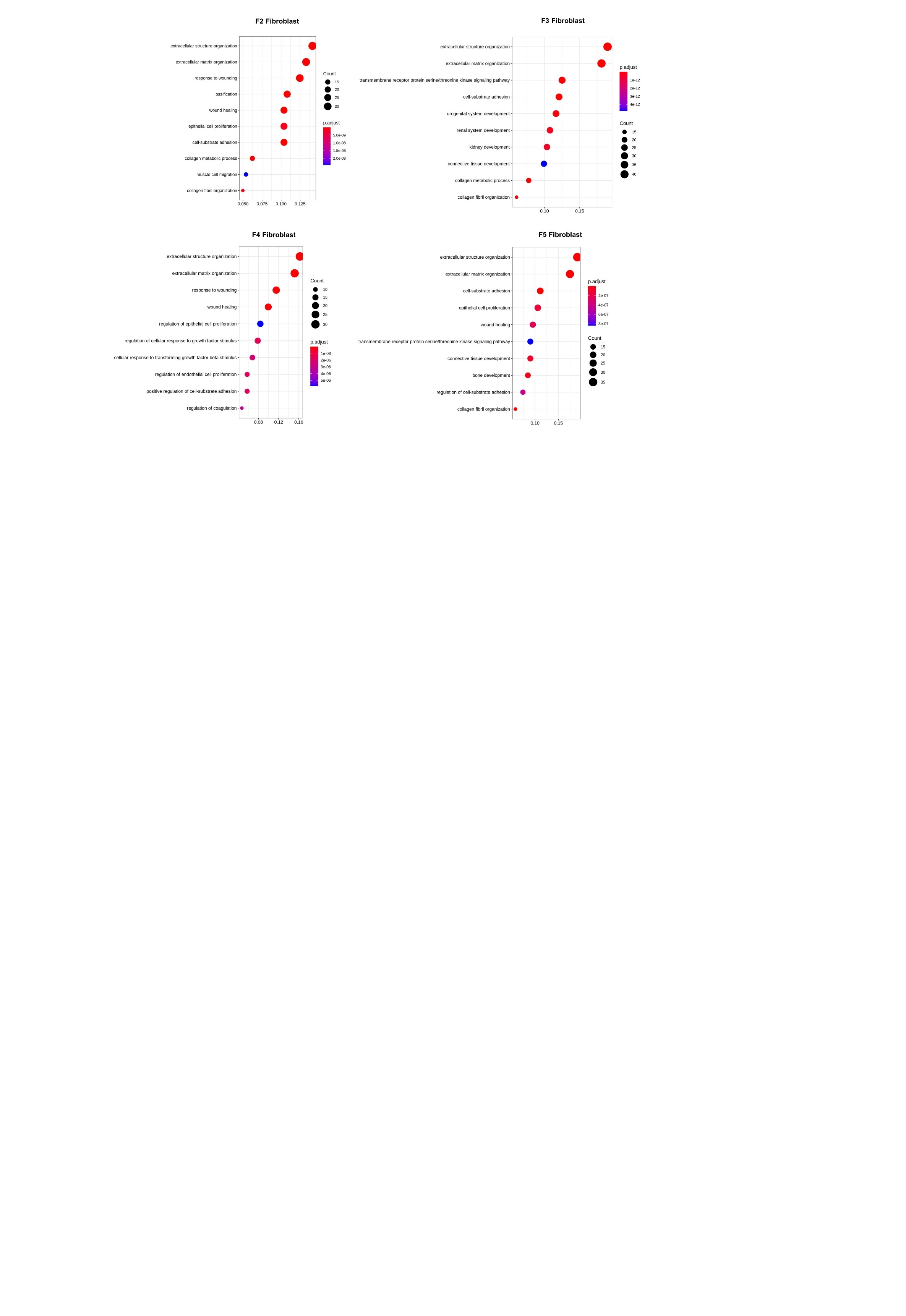


Fig. S8. The histogram of the biological processes of different fibroblast clusters. Gene Set Enrichment Analysis was used to determine p value using GO (biological process) database on Fibroblasts (FBs). We emphasized the novel GO terms related to fibroblast function.


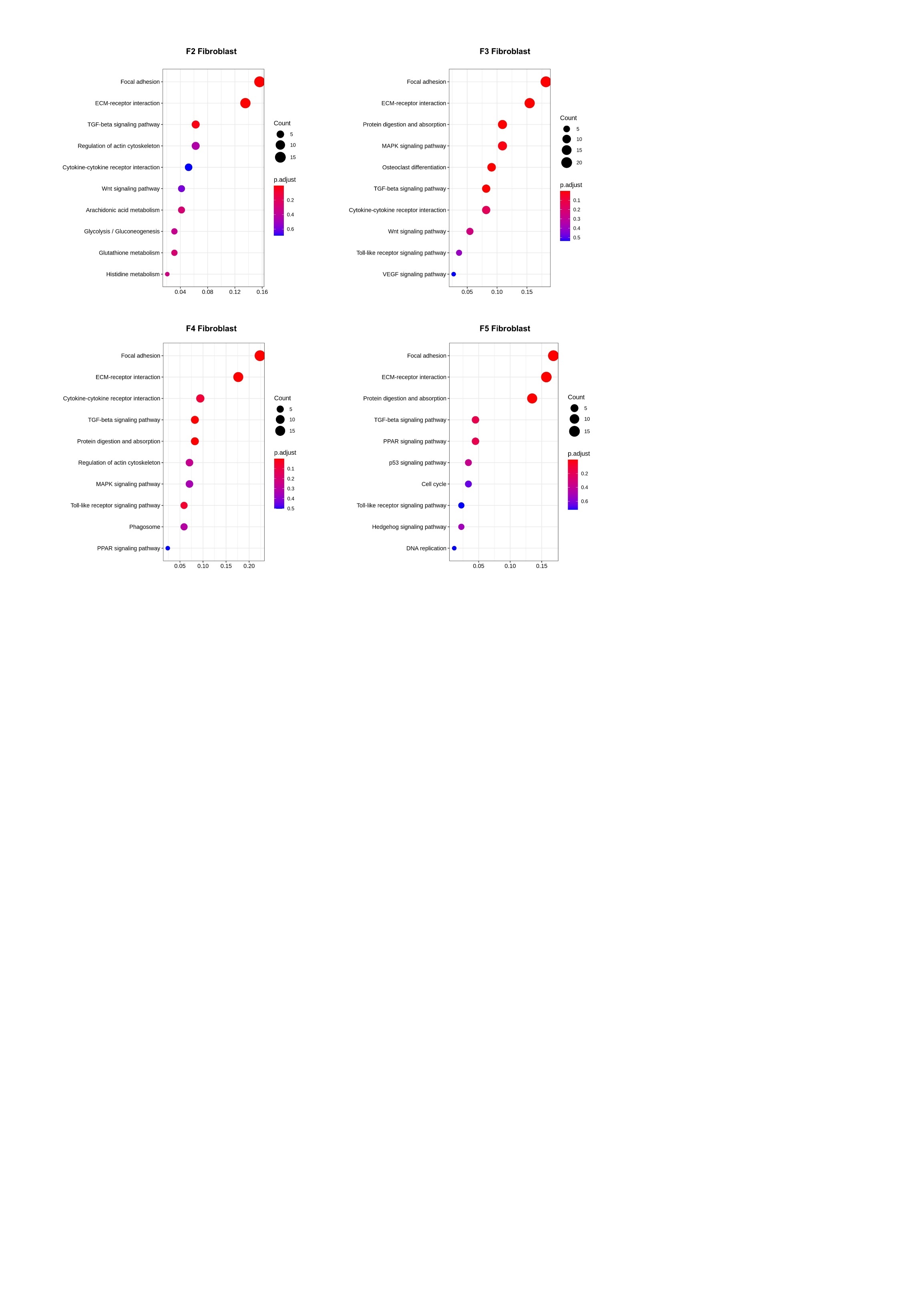


Fig. S9. The histogram of the biological processes of different fibroblast clusters. Gene Set Enrichment Analysis was used to determine p value using GO (biological process) database on Fibroblasts (FBs). We emphasized the novel KEGG terms related to fibroblast function.


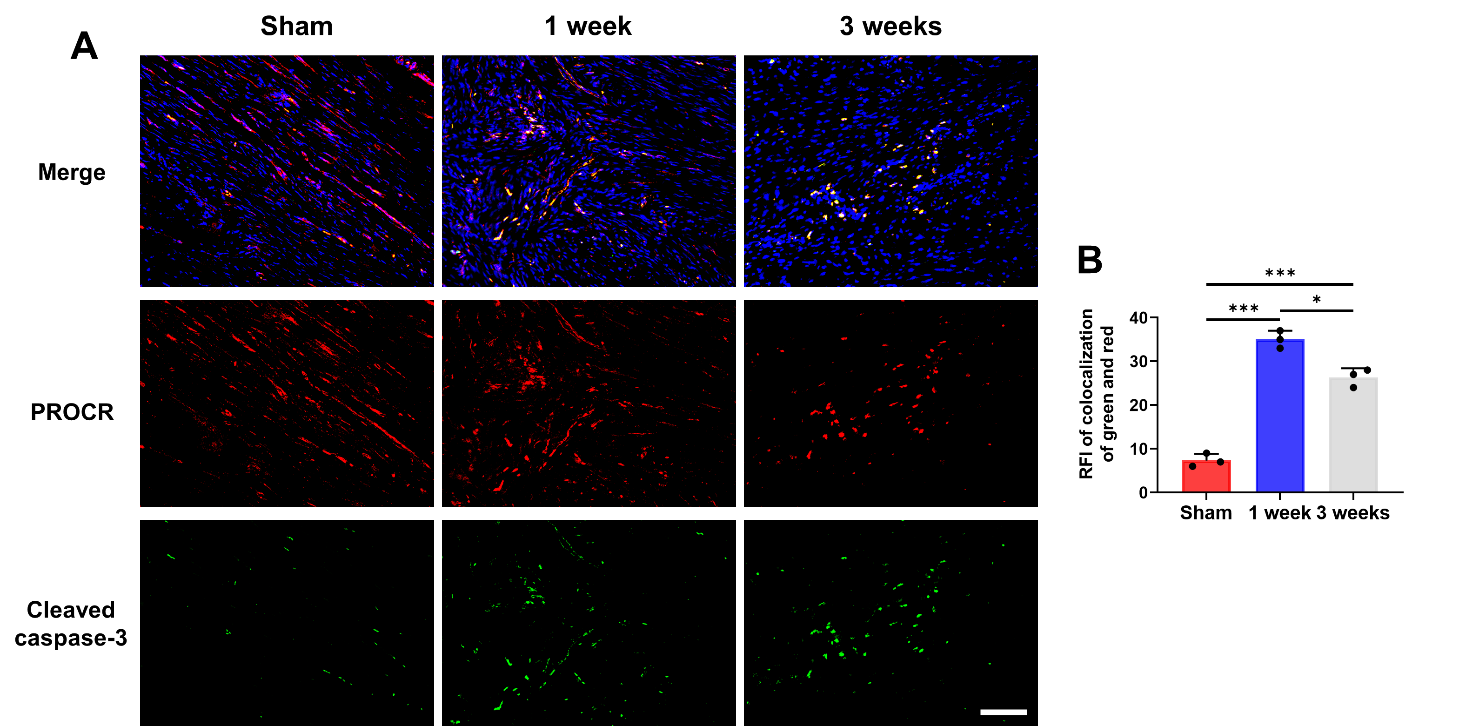


**Fig. S10.** Characterization of the apoptosis of the PROCR^+^ fibroblast in sham and HO tendons. (**A**) Immunofluorescence microscopy of Achilles tendons from the sham and HO groups after 1 and 3 weeks. PROCR, red; Cleaved caspase-3, green; DAPI, blue. Scale bar: 50 μm. (**B**) Quantitative analysis of the colocalization of the free green and red of Achilles tendons from sham and HO groups after 1 and 3 weeks (n = 3). RFI: relative fluorescence intensity. All data was presented as mean ± standard deviation. Statistical analyses are performed by one-way ANOVA with a post-hoc Tukey’s test. **P* < 0.05, ****P* < 0.001.


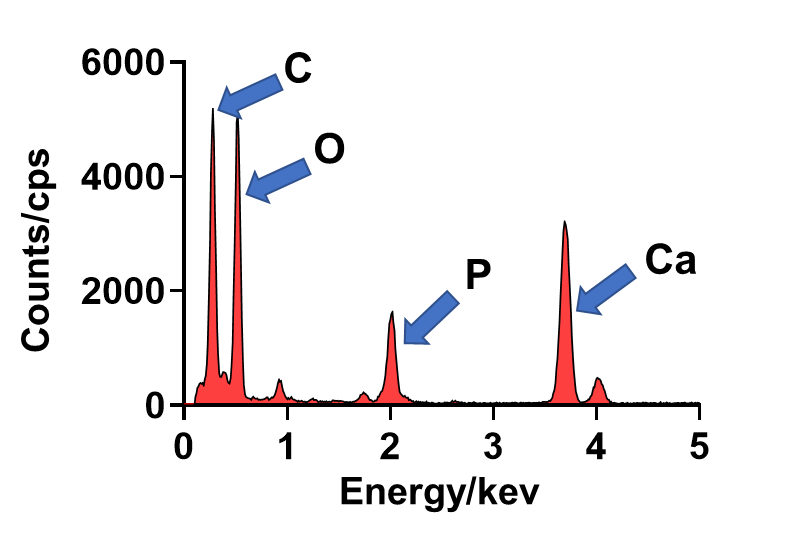


Fig. S11. EDS elemental analysis of the selected area of the calcified apoVs containing electron-dense granules.


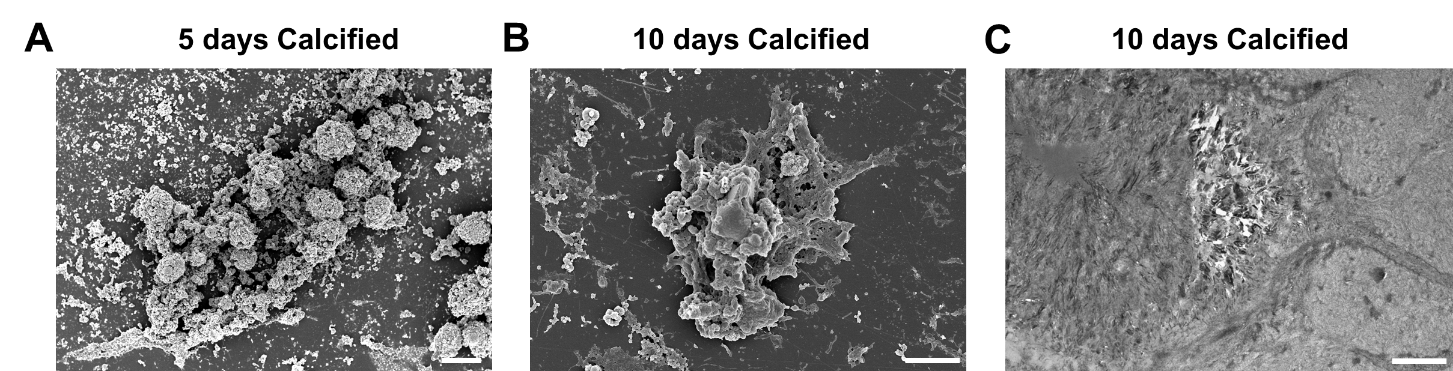


Fig. S12. Representative SEM and TEM images of the fibroblasts cultured in the calcified medium. (A) Representative SEM images of the fibroblasts cultured in the calcified medium for 5 days. Scale bar: 10 μm. (B) Representative SEM images of the fibroblasts cultured in the calcified medium for 10 days. Scale bar: 5 μm. (C) Representative TEM images of the fibroblasts cultured in the calcified medium for 10 days. Scale bar: 1 μm.


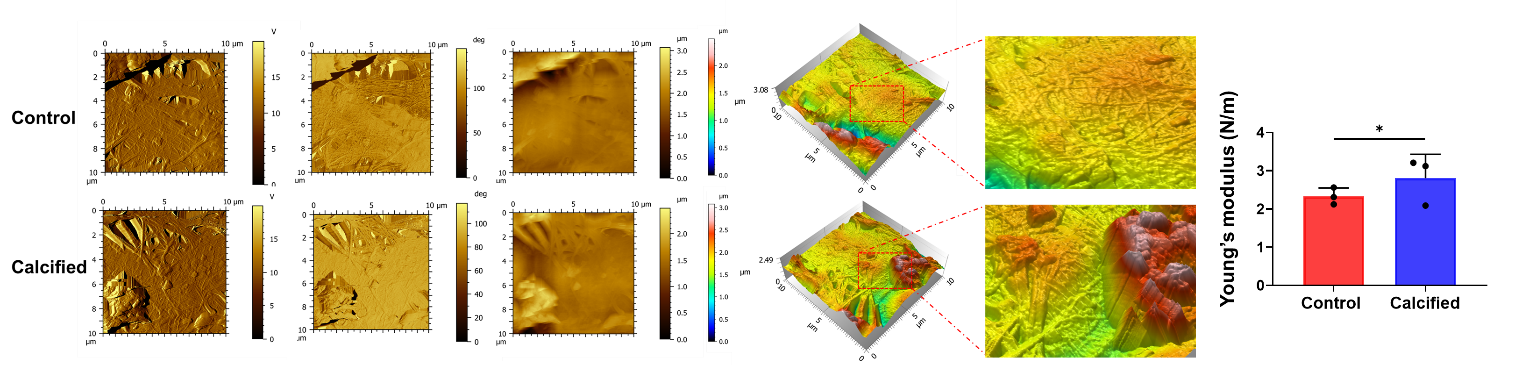


Fig. S13. Atomic force microscope measurements of the fibroblasts cultured in the control and calcified medium for 7 days. Left: the images of the fibroblasts under the AFM microscope. Right, AFM quantification between control and calcified group. Data were presented as means ± standard deviations (n = 3). Statistical analyses were performed by Student’s t test. **P* < 0.05.


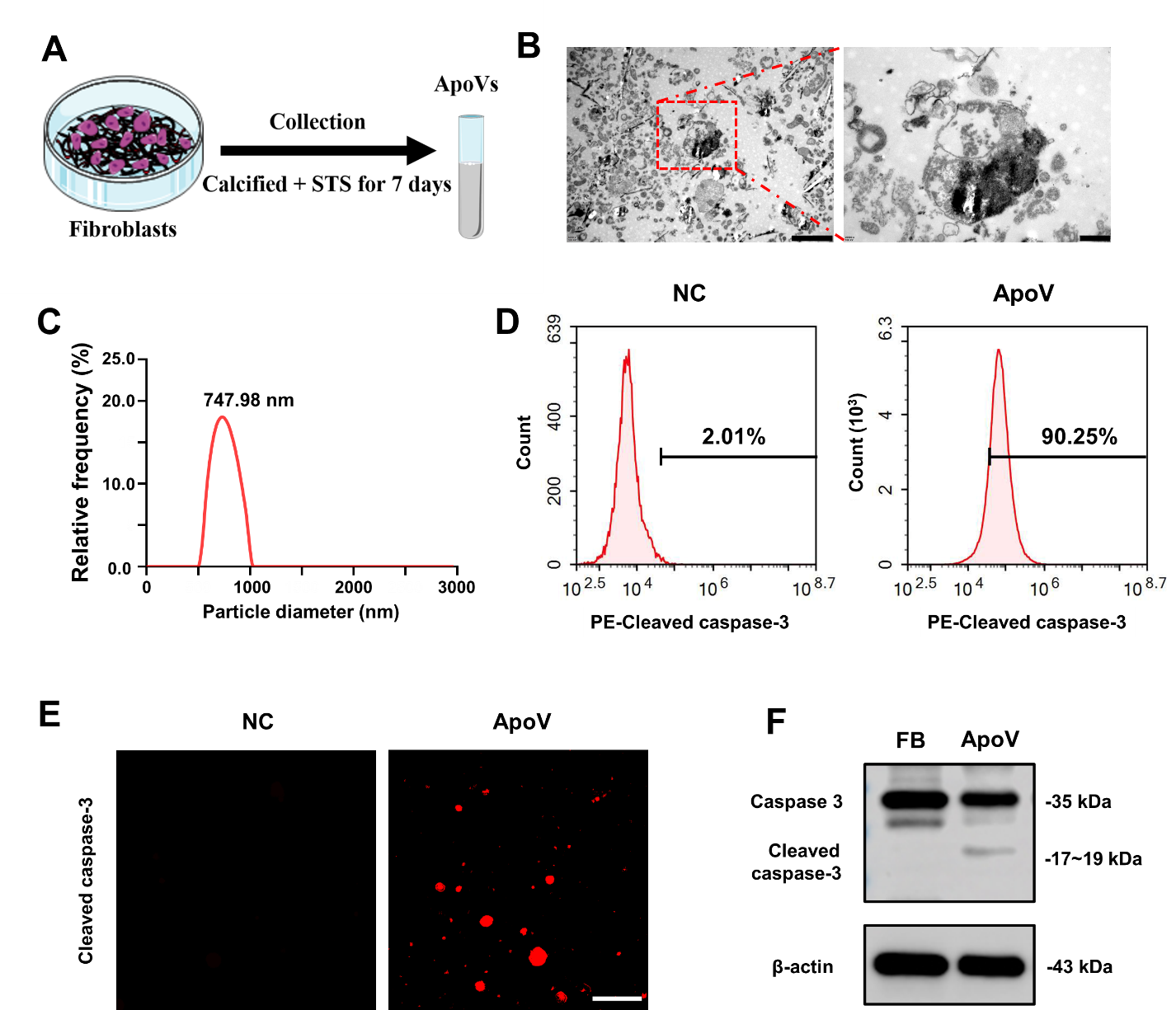


**Fig. S14**. The isolation and characterization of the apoVs. (**A**) Schematic representation of the isolation of the apoVs. STS, staurosporine. (**B**) TEM images of the apoVs from the fibroblasts. Scale bar: 2 μm. High magnification of the red rectangle showing the apoVs. Scale bar: 500 nm. (**C**) NTA of the apoVs from the fibroblasts. (**D**) Flow cytometric analysis of cleaved caspase-3 in the apoVs. (**E**) Representative confocal microscopy images of cleaved caspase-3 staining in the apoVs. Scale bars, 10 μm. (**F**) Western blotting analysis showing the presence of Caspase-3/Cleaved caspase-3 in fibroblasts (FB) and apoVs.


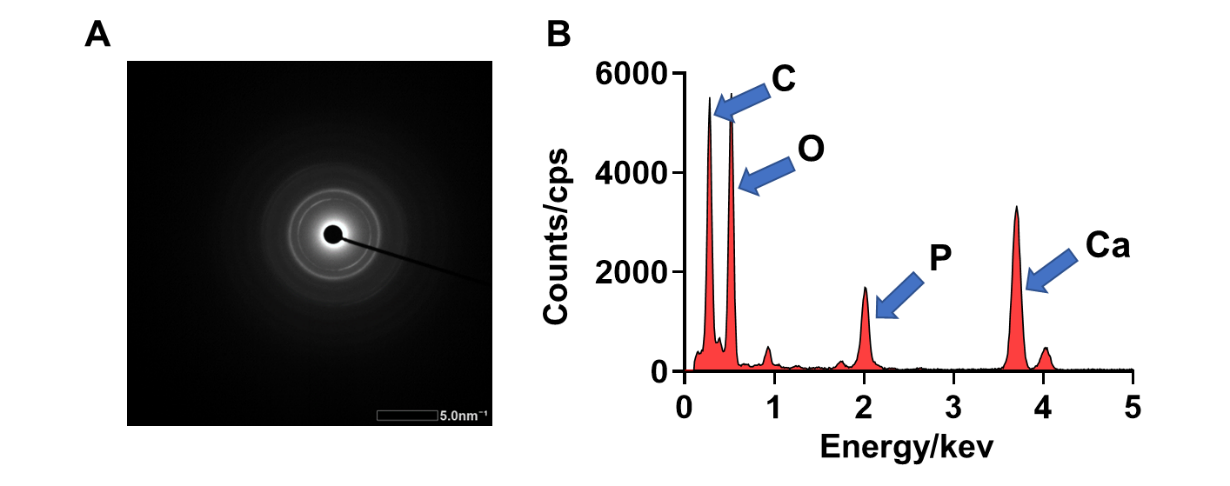


**Fig. S15.** Selected area electron diffraction (A) and element mapping (B) of the collagen scaffolds of the calcified apoVs group.


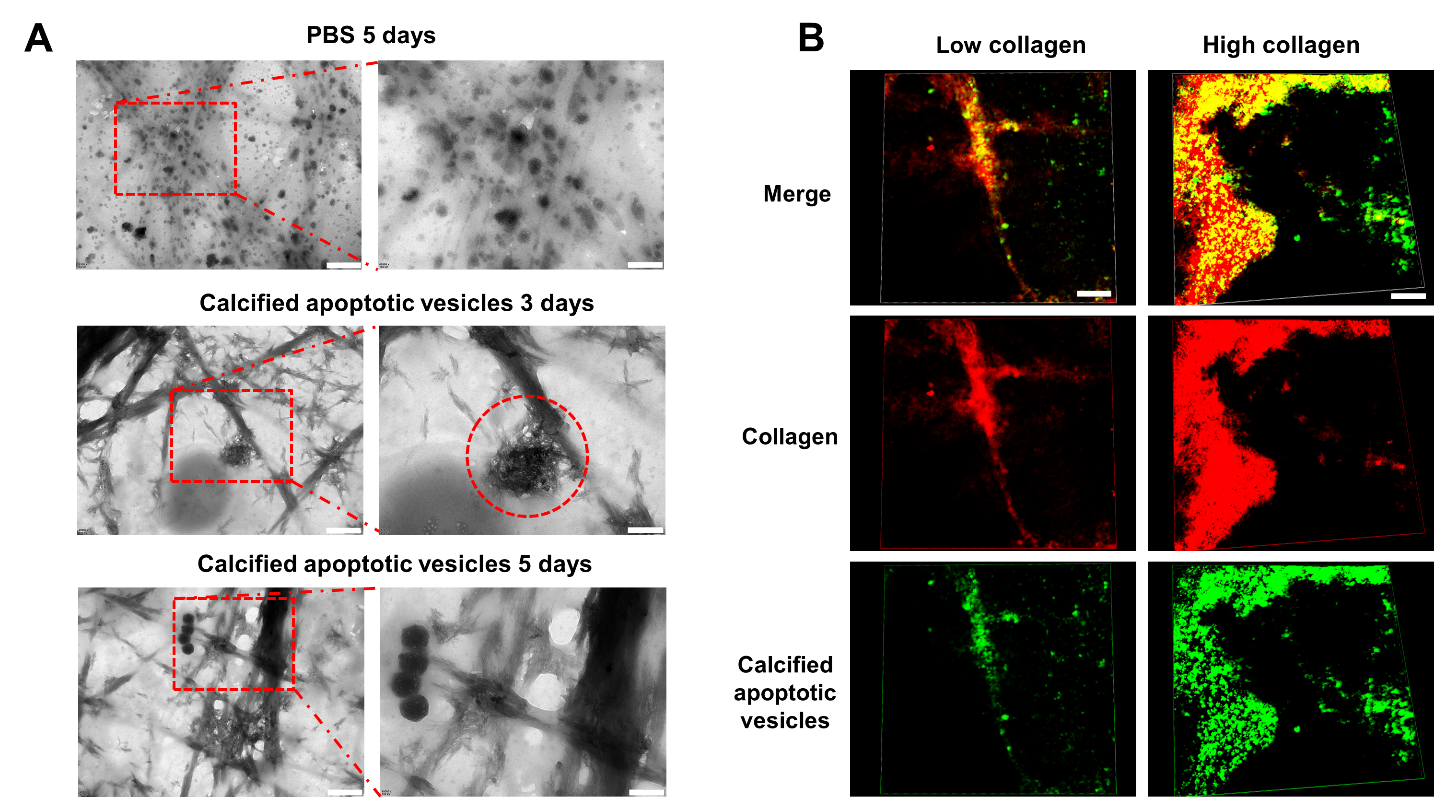


Fig. S16. (A) TEM images of the calcified apoVs incubated with the collagen I for 3 and 5 days. The calcified apoVs were collected from the fibroblasts cultured for 7 days in the calcified medium. Scale bar: 500 nm. High magnification of the red rectangle showing the calcified apoVs. Scale bar: 200 nm. (B) Immunofluorescence microscopy of the collagen I hydrogels after introduction of green-labeled calcified apoVs at 48 h. Scale bar: 5 μm.


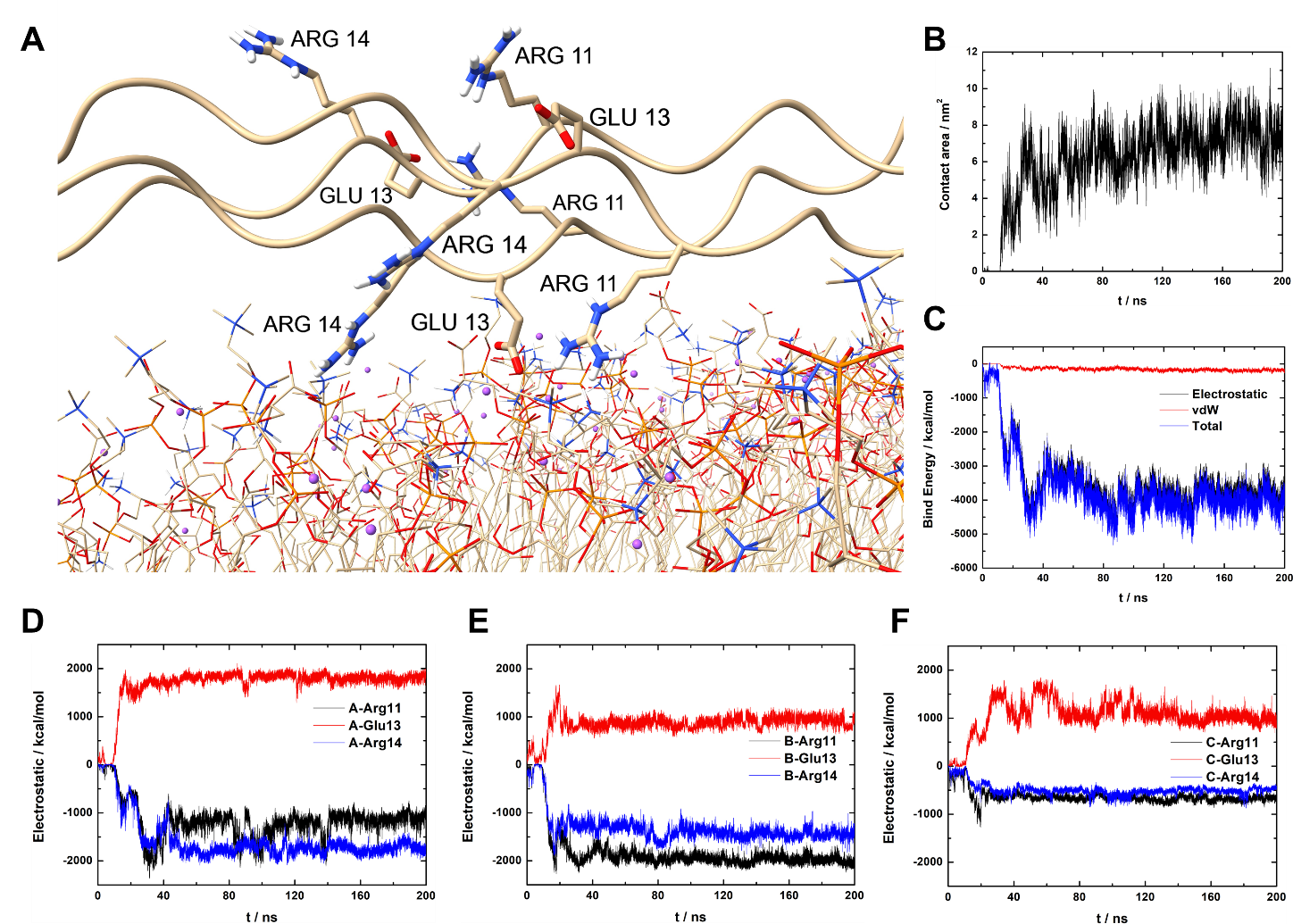


Fig. S17. Molecular dynamic (MD) simulation analysis between the calcified apoVs and collagen I. (A) Binding patterns analysis between the calcified apoVs and collagen I. (B) Contact area analysis between the calcified apoVs and collagen I (t = 200 ns). (C) Binding energy analysis between the calcified apoVs and collagen I (t = 200 ns). Electrostatic interaction is favorable for the calcified apoVs interaction with collagen fibrils. vdw, van der Waals energy. (D-F) Binding energy analysis of electrostatic interactions of the phospholipid membrane with the key binding residues of the A, B and C chains (t = 200 ns).


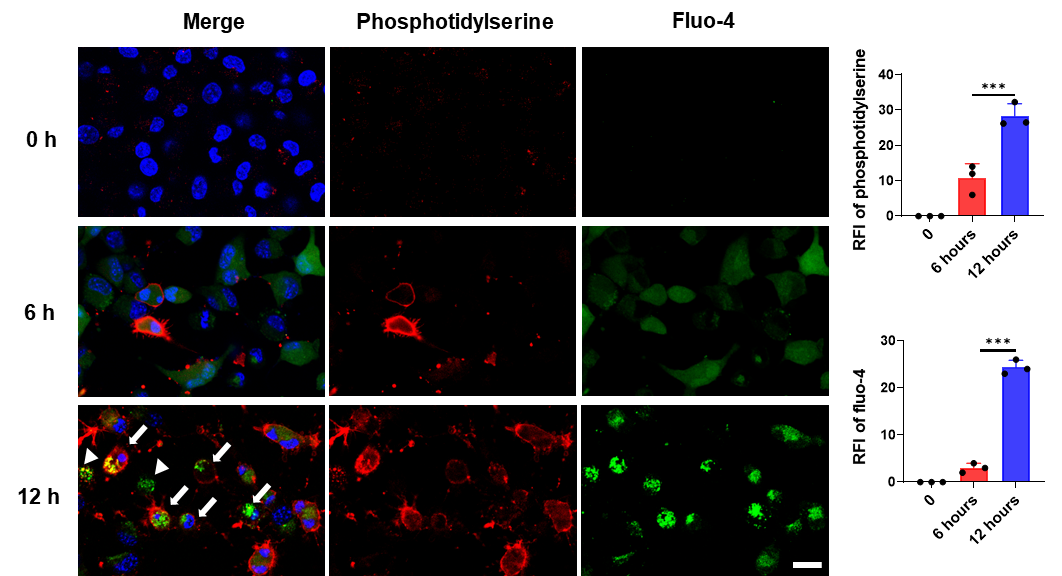


Fig. S18. Immunofluorescence microscope of the fibroblasts with high calcium and phosphorus concentrations medium for 6 and 12 hours. Phosphotidylserine (PS), marker for apoptosis, and Fluo-4, marker for calcium, were not detected in normal control group. After 6 hours, PS was clearly detectable and significantly up-regulated after 12 hours. Phosphotidylserine, red; Fluo-4, green; Dapi, blue. Scale bar, 5 μm. All data was presented as mean ± standard deviation. Statistical analyses are performed by one-way ANOVA with a post-hoc Tukey’s test. ****P* < 0.001.


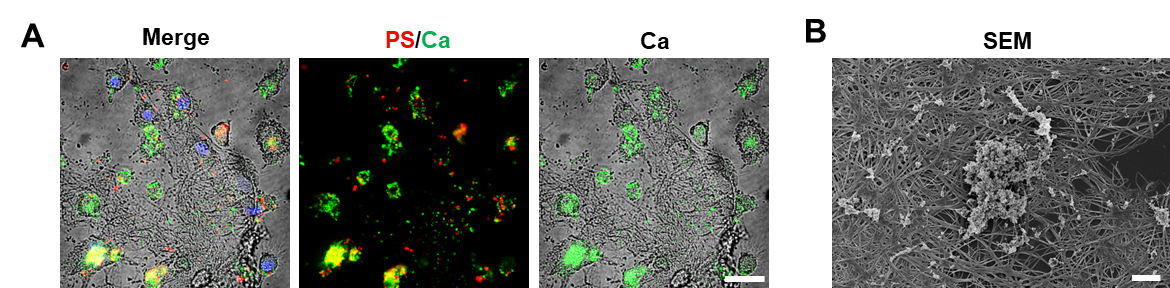


Fig. S19. The calcified apoVs continuously enriched calcium and released the calcification. (A) Immunofluorescence microscopy of the calcified apoVs cultured in the collagens with high calcium and phosphorus concentrations for 12 h. Phosphotidylserine, red; Fluo-4, green; Dapi, blue. Scale bar, 10 μm. (B) Representative SEM image based on (A). Scale bar, 2 μm.


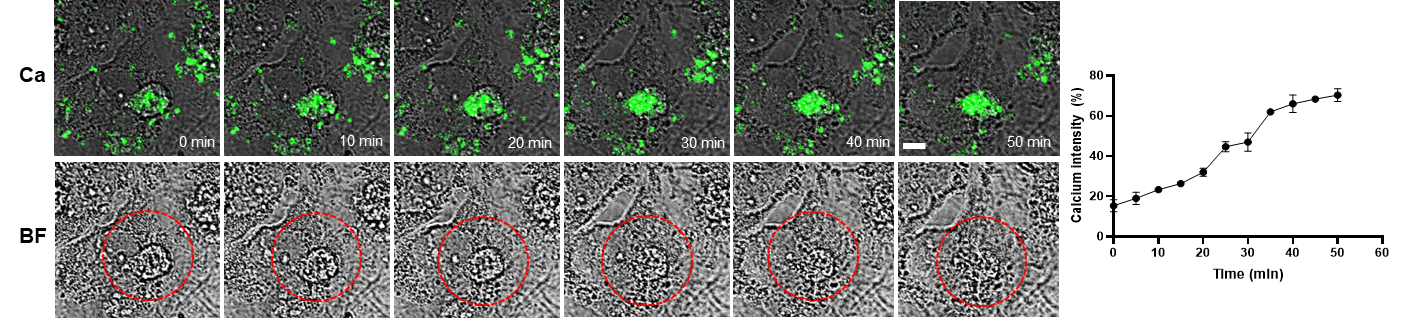


Fig. S20. Time-lapse images monitoring the calcified apoVs enriching calcium and the quantification of the intensity of Fluo-4 staining in the calcified apoVs. BF: Bright field.


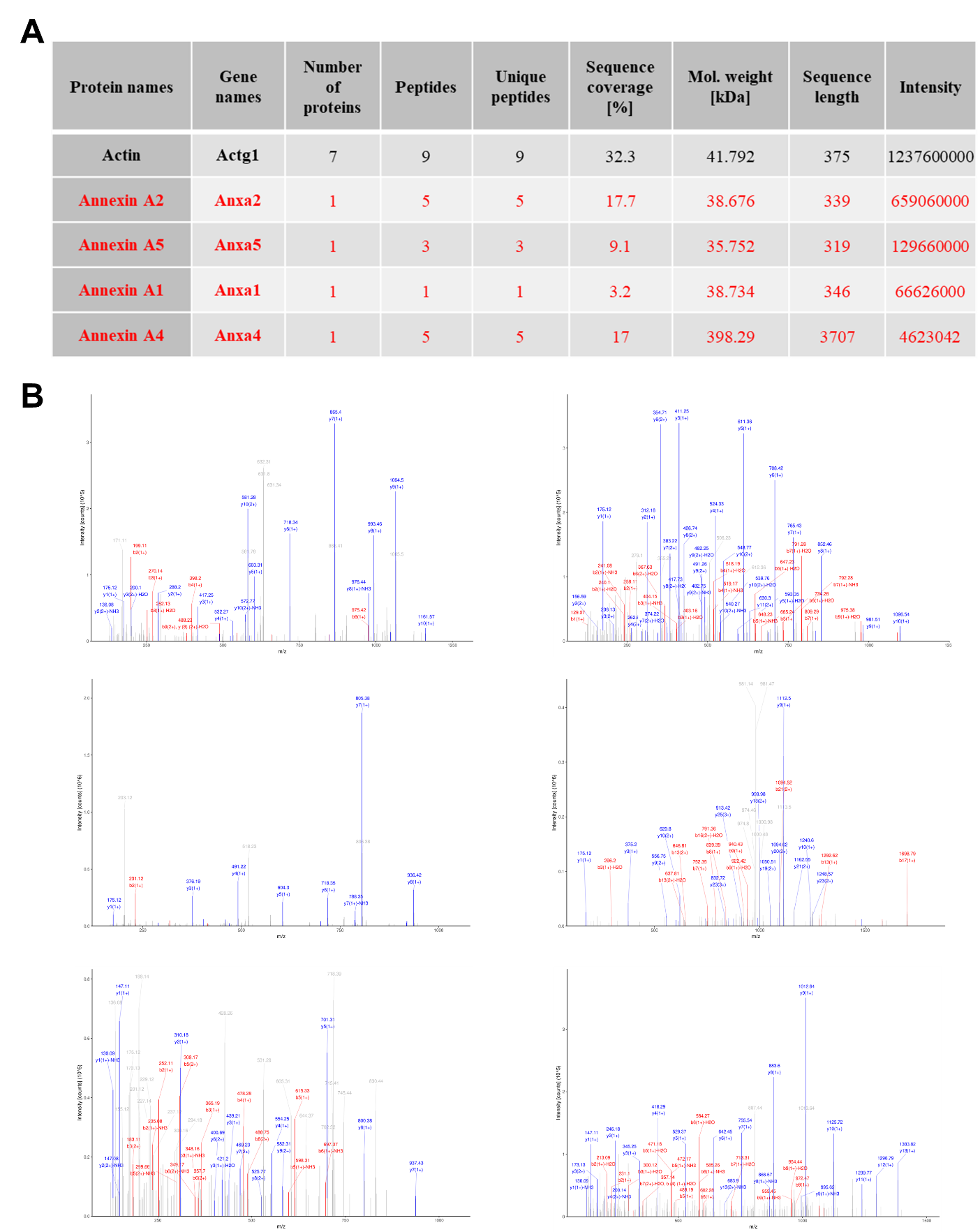


Fig. S21. The list of candidates and unique peptides of the top 5 scores in LC-MS/MS analysis of membrane protein of the calcified apoVs from the fibroblasts with high calcium and phosphorus concentrations.


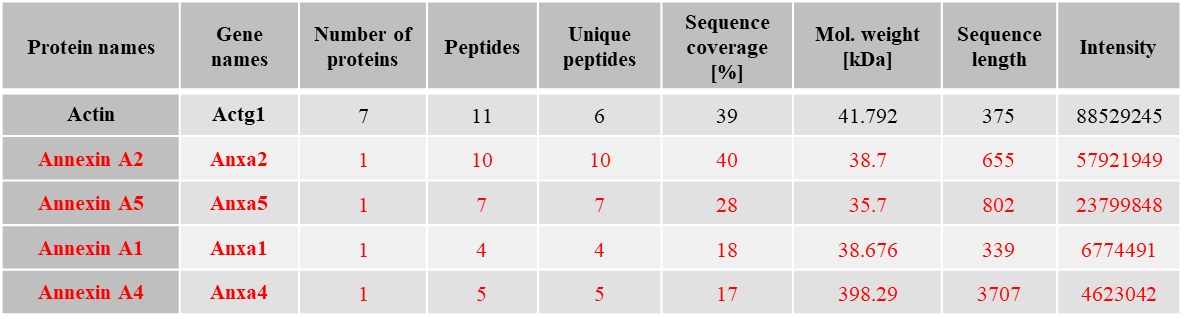


Fig. S22. List of candidates with top 5 scores in LC-MS/MS analysis of membrane protein of the calcified apoVs from the 1 week HO tendons.


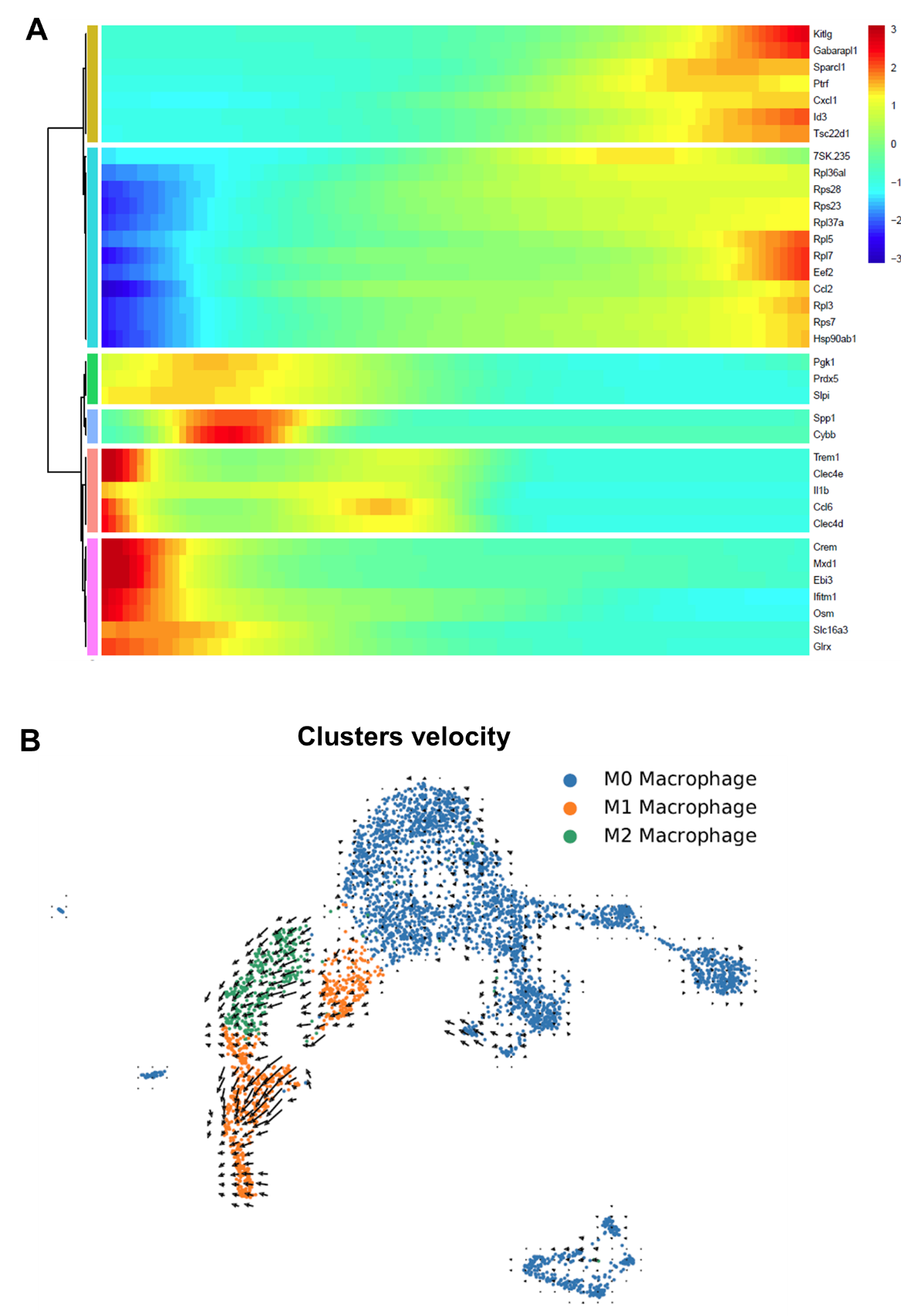


Fig. S23. The polarization of macrophage from M1 to M2. (A) Genes showing the change in expression along the cell differentiation trajectory by Monocle. Red indicates higher expression, blue indicate lower expression. (B) RNA velocity cell trajectory analysis of macrophages.


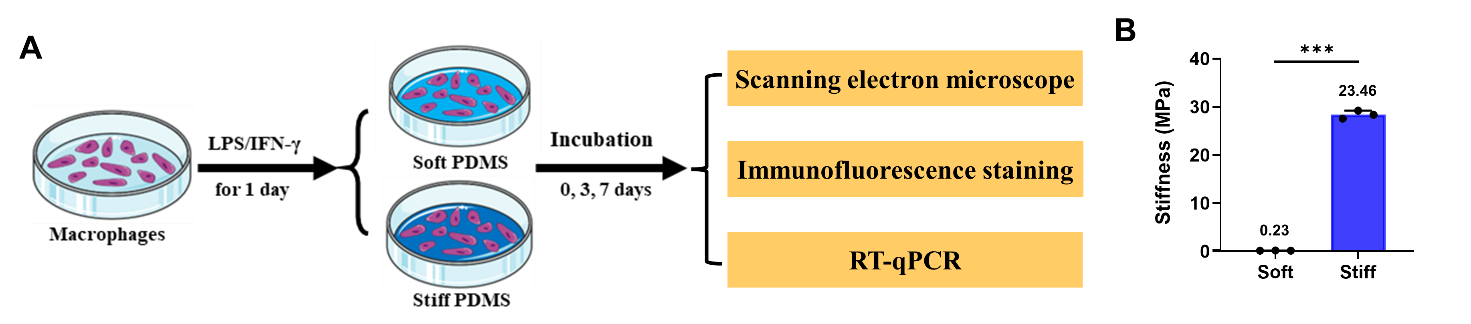


Fig. S24. The macrophage cultured in two kinds of PDMS substrates with different young’s moduli. (A) Schematic representation of the macrophage cultured in two kinds of PDMS substrates with different young’s moduli. (B) AFM quantification between two kinds of PDMS substrates. Data were presented as means ± standard deviations (n = 3). Statistical analyses were performed by Student’s t test. ****P* < 0.001.


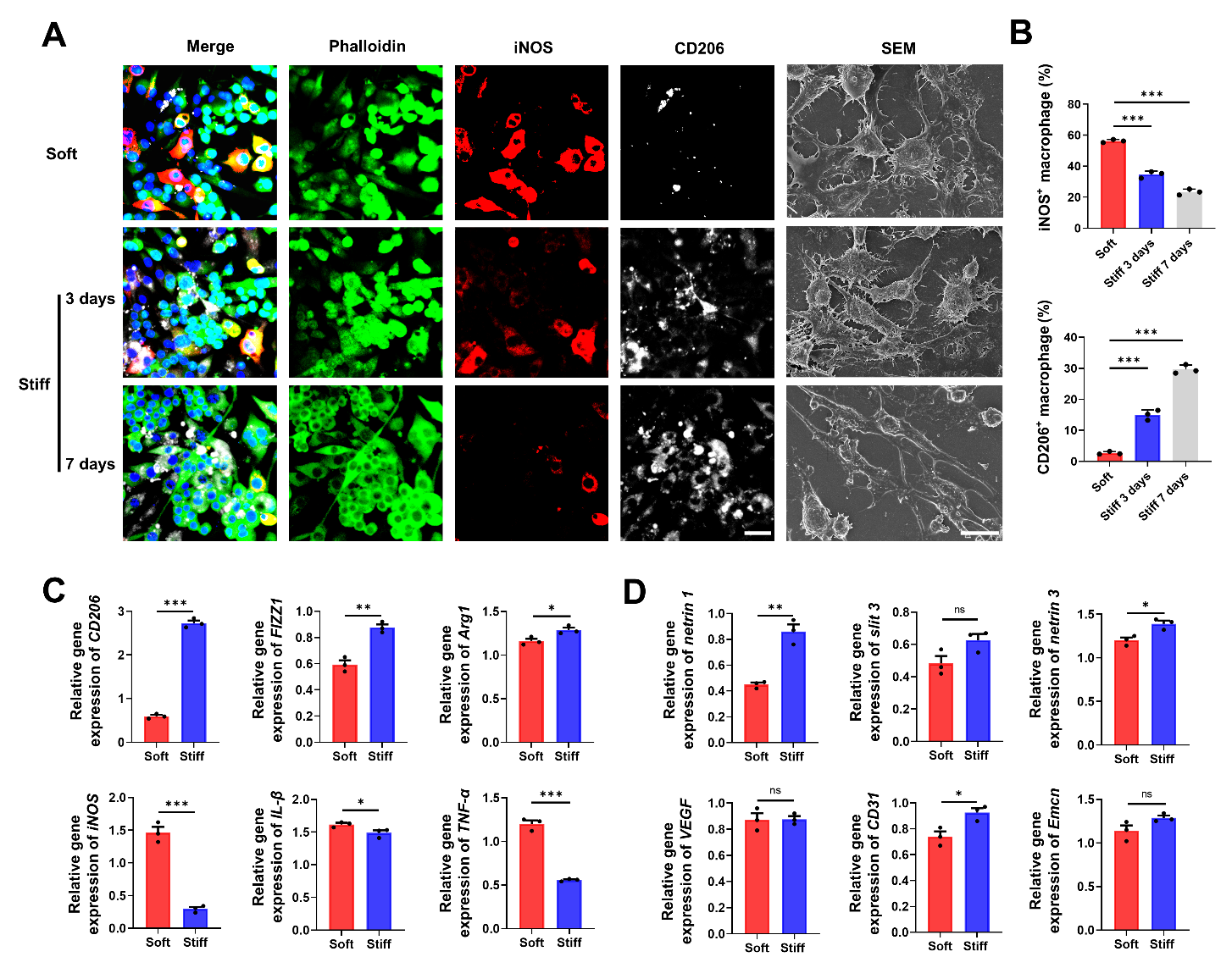


Fig. S25. Increased stiffness of ECM promoted the polarization of macrophage from M1 to M2. (A) Immunofluorescence microscopy of the macrophages in the different groups. Phalloidin, green; iNOS, red; CD206, white; DAPI, blue. Scale bar: 20 μm. (B) Quantitative analysis of iNOS^+^ cells and CD206^+^ cells in (A) (n = 3). (C, D) qRT-PCR analysis of the gene expression in two kinds of PDMS substrates with different young’s moduli (n = 3). Data was presented as means ± standard deviations. Statistical analyses were performed by one-way ANOVA with post-hoc Tukey’s test and Student’s t test. ns, no significance. **P* < 0.05, ***P* < 0.01, ****P* < 0.001.


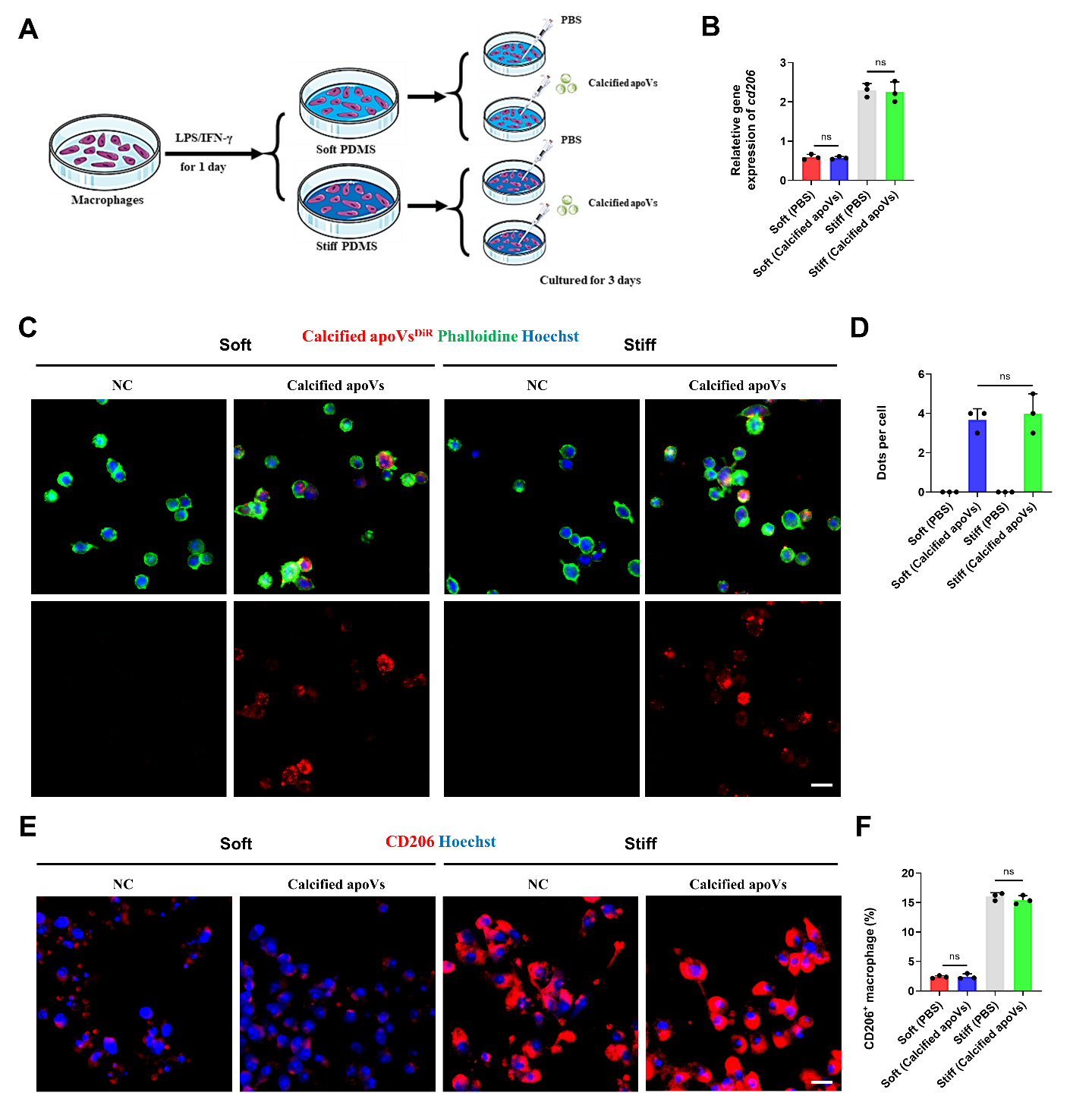


**Fig. S26**. The effect of the calcified apoVs on the polarization of macrophages to M2. **(A)** Schematic representation of the macrophage cultured in two kinds of PDMS substrates with the calcified apoVs. **(B)** qRT-PCR analysis of the gene expression of the macrophages in four groups (n = 3). **(C)** Immunofluorescence staining of calcified apoVs with DiR (red), phalloidine (green) and Hoechst (blue) after the cells were incubated with calcified apoVs for 3 days. Scale bar, 2 μm. **(D)** Quantitative analysis of the calcified apoVs (red dots) in the macrophages (n = 3). **(E)** Immunofluorescence microscopy of the macrophages in the different groups. CD206, red; Hoechst, blue. Scale bar: 2 μm. **(F)** Quantitative analysis of CD206+ cells in (E) (n = 3). Data was presented as means ± standard deviations. Statistical analyses were performed by one-way ANOVA with post-hoc Tukey’s test. ns, no significance.

| Genes | Description | | | Primer sequences |
| --- | --- | --- | --- | --- |
| *VEGF* | m-*VEGF*-F | | 5’- ACATTGGCTCACTTCCAGAAACAC-3’ | |
|  | m-*VEGF-*R | | 5’- TGGTTGGAACCGGCATCTTTA-3’ | |
| *iNOS* | h- *iNOS*-F | | 5’- CAAGCTGAACTTGAGCGAGGA-3’ | |
|  | h- *iNOS*-R | | 5’- TTTACTCAGTGCCAGAAGCTGGA-3’ | |
| *IL-1β* | h- *IL-1β*-F | | 5’- TCCAGGATGAGGACATGAGCAC-3’ | |
|  | h- *IL-1β*-R | | 5’- GAACGTCACACACCAGCAGGTTA-3’ | |
| *TNF-α* | h- *TNF-α* -F | | 5’- ACTCCAGGCGGTGCCTATGT-3’ | |
|  | h- *TNF-α* -R | 5’- GTGAGGGTCTGGGCCATAGAA-3’ | | |
| *CD206* | h- *CD206*-F | | 5’- AGCTTCATCTTCGGGCCTTTG-3’ | |
|  | h- *CD206*-R | | 5’- GGTGACCACTCCTGCTGCTTTAG-3’ | |
| *Arg1* | h- *Arg1*-F | | 5’- AGCTCTGGGAATCTGCATGG-3’ | |
|  | h- *Arg1*-R | | 5’- ATGTACACGATGTCTTTGGCAGATA-3’ | |
| *FIZZ1* | h- *FIZZ1*-F | | 5’- CAGCTGATGGTCCCAGTGAA-3’ | |
|  | h- *FIZZ1*-R | | 5’- CAAGCACACCCAGTAGCAGTC-3’ | |
| *CD31* | h- *CD31*-F | | 5’- TGGTTGTCATTGGAGTGGTC-3’ | |
|  | h- *CD31*-R | | 5’- TTCTCGCTGTTGGAGTTCAG-3’ | |
| *Emcn* | h- *Emcn*-F | | 5’- CCAGTCTTCTCACCAGTCTACAC-3’ | |
|  | h- *Emcn*-R | | 5’- TCACAGTTCCTCCTAGCAAAGTC-3’ | |
| *Slit3* | h- *Slit3*-F | | 5’- AGACCCTGAACCTGGTGGTAGAC-3’ | |
|  | h- *Slit3*-R | | 5’- TCTGCACCCTGGCGTAAGG-3’ | |
| *netrin 1* | h- *netrin 1*-F | | 5’- GTTCGGCGACGAGAACGAA-3’ | |
|  | h- *netrin 1*-R | | 5’- TGTGCCTACAGTCACACACCAGA-3’ | |
| *netrin 3* | h- *netrin 3*-F | | 5’- AGGTCGGCCAATCGCGT-3’ | |
|  | h- *netrin 3*-R | | 5’- CAGGGGCACTGTGAGGGTTAC-3’ | |
| *Gapdh* | h-*Gapdh* -F | | 5’-TGTGTCCGTCGTGGATCTGA-3’ | |
|  | h-*Gapdh*-R | | 5’-TTGCTGTTGAAGTCGCAGGAG-3’ | |

**Table S1**. Primers for qRT-PCR.

**Supplementary Materials and Methods**

*Micro-CT analyses*

Achilles tendons with calcaneus and lower tibias from rats in the HO and sham groups were fixed overnight in 4% paraformaldehyde and analyzed by micro-CT (Inveon micro-CT system Siemens AG, Germany). The scanner was set at a voltage of 80 kV at 500 mA and a resolution of 10 μm per pixel. Two-dimensional slices with 10 μm isotropic resolution were generated with a three dimensional (3D) image reconstruct based on the scanned information using the Inveon Research Workplace software (Siemens Medical Solutions USA, Inc., Hoffman Estates, IL, USA). A region of interest was positioned in the injury site and the bone mineral density (BMD), bone volume/tissue volume (BV/TV) and bone surface/ bone volume (BS/BV) were measured.

*Histologic and immunohistochemistry evaluation*

After fixation, the tissues were processed for paraffin embedding to collect 4-μm-thick sections. After deparaffinized and rehydrated in xylene and a declining graded series of ethanol, the sections were stained with hematoxylin and eosin (G1120, Solarbio, CN) and observed by microscope (Leica, DM 2500, Wetzlar, Germany).

For alizarin red S staining, the sections were stained with alizarin red S (40 mmol/L, pH 4.2; MilliporeSigma, Burlington, USA) for 20 min. The nuclei were counterstained with DAPI (Invitrogen).

For immunohistochemical staining, the sections were incubated with goat serum to block special sites and then individually incubated with cleaved caspase-3 (#40500, Signalway Antibody, College Park, Maryland, USA) after deparaffinization, hydration and blockage of endogenous peroxidase. Followed by secondary antibody anti-rabbit IgG (Kit-5010, MXB, CN) incubation for 1 hour at 37 °C, the colorization developed with DAB solution (DAB-4033, MXB, CN) and counterstained in hematoxylin.

For immunofluorescence staining, the primary antibodies used were: PROCR (bs-9506R, Bioss, Beijing, China), PDGF receptor-α (ab96569, Abcam, Cambridge, UK), cleaved caspase-3 (#40500, Signalway Antibody). The sections were incubated with secondary antibody (US Everbright Inc., Suzhou, China). After incubation, the sections were washed with PBS and the nuclei were counterstained with DAPI (Invitrogen).

For TUNEL staining, the terminal deoxynucleotidyl transferase-mediated deoxyuridine triphosphate nickend labeling staining was performed with the detection kit (11684795910, Roche, Mannheim, Germany) for *in situ* detection of fibroblasts in tendon. The percentage of TUNEL-positive cells was calculated from the number of total cells using the image-pro Plus 6.0 (Media Cybernetics, Rockville, MD).

*Scanning electron microscope (SEM) and energy-dispersive X-ray spectroscopy*

2.5% glutaraldehyde in phosphate buffer (0.01 M, pH = 7.4) was used to fix tendons. Specimens were then dehydrated with an ascending series of ethanol and treated with hexamethyldisilane (Electron Microscopy Sciences, Hatfield, PA, USA). Field-emission scanning electron microscope (FE-SEM, S-4800, Hitachi, Tokyo, Japan) operated at 5 kV was used to observe the samples. The mineral elemental composition in the tendons was characterized by using energy-dispersive X-ray spectroscopy (Element EDS System, Ametek, Berwyn, PA, USA).

*Elemental mapping*

Elemental mapping was performed using a scanning TEM (STEM) equipped with EDS. Specimens used for elemental mapping were not post-fixed with osmium tetroxide to avoid interference during EDS. Unstained thin sections were examined with a Technai G2 STEM (FEI, Hillsboro, USA) at 200 kV. Elemental mapping and selected area electron diffraction (SAED) were conducted using an INCA X-sight detector (Oxford Instruments, Abingdon, UK). Mappings were acquired with the FEI TIA software using a spot dwell time of 300 ms with drift correction performed after every 30 images.

*Calcein fluorescent labeling and histomorphometrical analysis*

The process of calcification was assessed using a calcein fluorescent labeling method. 8 week old rats received achillotenotomy and an injection of calcein green. Briefly, mice were intraperitoneally injected with 20 mg/kg of calcein (Sigma-Aldrich) in a 2% sodium bicarbonate solution after achillotenotomy (Zhang et al., 2013). The rats were euthanized 1 and 3 weeks respectively. The tissues were fixed overnight in 4% paraformaldehyde and dehydrated with 30% saccharose, and finally embedded in OCT (Leica, Wetzlar, Germany) to collect 6-μm-thick sections. Those sections were subsequently stained with DAPI (Invitrogen) for fluorescence labeling observation under confocal laser scanning microscope (CLSM, Leica). Excitation/emission wavelengths of chelating fluorochromes were used 488/517 nm for calcein fluorescent labeling (green).

*Fourier Transform Infrared Spectroscopy*

The spotlight 400 m-FTIR imaging system (PerkinElmer, Inc., Waltham, MA, USA) was used to analyze the sections from samples of tendons in sham and HO groups. The FTIR spectrometer was equipped with a liquid nitrogen-cooled 16-element linear array Mercury Cadmium Telluride (MCT) detector. The resolution of spectral acquisition was 4 cm^-1^, ranging from 650 to 4000 cm^-1^, and 2400 scans were used for each sample. Spectrum software (PerkinElmer, Inc.) was used to obtain the absorption wavelengths.

*Tissue dissociation and cell purification*

Tissues were transported in sterile culture dish with 10 mL 1X Dulbecco's Phosphate-Buffered Saline (DPBS; Thermo Fisher, Cat. no. 14190144) on ice to remove the residual tissue storage solution, then minced on ice. We used dissociation enzyme 0.25% Trypsin (Thermo Fisher, Cat. no. 25200-072) and 10 μg/mL DNase I (Sigma, Cat. no. 11284932001) dissolved in PBS with 5% Fetal Bovine Serum (FBS; Thermo Fisher, Cat. no. SV30087.02) to digest the tissues. The tissues were dissociated at 37 ℃ with a shaking speed of 50 rpm for 40 min. We repeatedly collected the dissociated cells at interval of 20 min to increase cell yield and viability. Cell suspensions were filtered using a 40 um nylon cell strainer and red blood cells were removed by 1X Red Blood Cell Lysis Solution (Thermo Fisher, Cat. no. 00-4333-57). Dissociated cells were washed with 1x DPBS containing 2% FBS. Cells were stained with 0.4% Trypan blue (Thermo Fisher, Cat. no. 14190144) to check the viability on Countess® II Automated Cell Counter (Thermo Fisher).

*10 x library preparation and sequencing*

Beads with unique molecular identifier (UMI) and cell barcodes were loaded close to saturation, so that each cell was paired with a bead in a Gel Beads-in-emulsion(GEM). After exposure to cell lysis buffer, polyadenylated RNA molecules hybridized to the beads. Beads were retrieved into a single tube for reverse transcription. On cDNA synthesis, each cDNA molecule was tagged on the 5’ end (that is, the 3’ end of a messenger RNA transcript) with UMI and cell label indicating its cell of origin. Briefly, 10X beads that were then subject to second-strand cDNA synthesis, adaptor ligation, and universal amplification. Sequencing libraries were prepared using randomly interrupted whole-transcriptome amplification products to enrich the 3’end of the transcripts linked with the cell barcode and UMI. All the remaining procedures including the library construction were performed by Shanghai Biotechnology Corporation (CG000206 RevD). Sequencing libraries were quantified using a high sensitivity DNA Chip (Agilent) on a Bioanalyzer 2100 and the Qubit High Sensitivity DNA assay (Thermo Fisher Scientific). The libraries were sequenced on NovaSeq6000 (Illumina) using 2x150 chemistry.

*Single cell RNA-seq data processing*

Beads were processed using the Cell Ranger 2.1.0 pipeline with default and recommended parameters. FASTQs generated from Illumina sequencing output were aligned to the mouse genome, version GRCm38, using the STAR algorithm. Next, Gene-Barcode matrices were generated for each individual sample by counting UMIs and filtering non-cell associated barcodes. Finally, we generate a gene-barcode matrix containing the barcoded cells and gene expression counts. This output was then imported into the Seurat (v2.3.0) R toolkit for quality control and downstream analysis of our single cell RNA seq data (Satija et al., 2015). All functions were run with default parameters, unless specified otherwise. We excluded cells with fewer than 200 or more than 6000 detected genes (where each gene had to have at least one UMI aligned in at least three cells). The expression of mitochondria genes was calculated using Percentage Feature Set function of the seurat package (Satija et al., 2015). The normalized data (Normalize Data function in Seurat package) was performed for extracting a subset of variable genes. Variable genes were identified while controlling for the strong relationship between variability and average expression. Next, we integrated data from different samples after identifying ‘anchors’ between datasets using Find Integration Anchors and Integrate Data in the seurat package (Stuart et al., 2019). Then we performed principal component analysis (PCA) and reduced the data to the top 30 PCA components after scaled the data. We visualized the clusters on a 2D map produced with t-distributed stochastic neighbor embedding (t-SNE) (Maaten et al., 2008).

*Identification of cell types and subtypes by nonlinear dimensional reduction (t-SNE)*

Cells were clustered using graph-based clustering of the PCA reduced data with the Louvain Method (Blondel et al., 2008) after computing a shared nearest neighbor graph (Satija et al., 2015). For sub-clustering, we applied the same procedure of scaled, dimensionality reduction and clustering to the specific set of data (usually restricted to one type of cell). For each cluster, we used the Wilcoxon Rank-Sum Test to find significant deferentially expressed genes comparing the remaining clusters. Single R (Aran et al., 2019) and known marker genes were used to identify cell type.

*Apoptotic marker detection of the calcified apoVs*

The calcified apoVs were characterized by western blotting using anti-Caspase-3 (#27525, Signalway Antibody) and anti-β-Actin (#52901, Signalway Antibody) antibodies. Additionally, apoVs were stained with cleaved caspase-3 (#40500, Signalway Antibody) and PE–conjugated Goat Anti-Rabbit IgG(H+L)(SA00008-2, Proteintech Group, Inc, Chicago, IL, USA), and followed by observation under a confocal microscope (FV1000, Olympus, Japan) and detection via a flow cytometer (CytoFLEX, Beckman Coulter, Brea, CA).

*Cell culture*

L929 fibroblasts (mouse fibroblast cell line) purchased from Procell were seeded in DMEM medium (PM150421, Pricella, CN) supplemented with 10% fetal bovine serum (PC-00001; PlantChemMed Biology Co., Ltd ShangHai, China) and 1% penicillin/streptomycin (PC-86115; PlantChemMed Biology Co.) in 5% CO_2_ at 37℃. To collect the calcified apoVs from the supernatant, the fibroblasts were seeded at a density of 10 ×10^5^/mL, and then cultured in calcified medium (10 nM dexamethasone, 100 μM L-ascorbic acid, 10 mM β-glycerophosphate, 1.1 mM calcium chloride and 100 nM staurosporine)(MilliporeSigma) for 7 days. The supernatant was collected and subsequently centrifuged at 800 × *g* for 10 min and 2,000 × *g* for 30 min. The apoVs were isolated from the supernatant by centrifugation at 7,000 × *g* for 30 min and then washed twice using filtered PBS.

For bone marrow-derived macrophages (BMDMs) preparation, both ends of the femur and tibia were cut and flushed with a syringe filled with FACS Buffer (HBSS + 10% FCS) to extrude bone marrow cells into a sterile petri dish. Then the cells were cultured in cRPMI medium with 50 ng/ml M-CSF for 6 days for BMDMs.

*PDMS substrates*

PDMS substrates were prepared as described previously (Xu et al., 2021). A polydimethylsiloxane substrate SYLGARDTM 184 Silicone Elastomer Kit (PDMS, Midland, MI, USA), comprising 2 components, a silicone elastomer and a silicone elastomer curing agent, were used. For the preparation of 15:1 (higher stiff substrate) and 45:1 (soft substrate) PDMS gels, silicone elastomer and the curing agent were thoroughly mixed together. The mixture was placed into a mold and then cross-linked on a heater at 60 ℃ for 12 h. PDMS substrate gels were next sterilized using UV overnight, and then the cells were seeded on the gels. The stiffness of the mixtures was measured using AFM.

*ApoVs labeling and cellular uptake assay*

The calcified apoVs were labeled with DiR fluorescent (PC-90202, PlantChemMed Biology Co.). Then, the apoVs were suspended in PBS. We cocultured the DiR labeled apoVs (500 μg per dish) with macrophages. After removal of unbound DiR, the stained apoVs were resuspended in PBS and underwent centrifugation, after which the supernatant was used as the negative control and injected. At the indicated time, we stained the cytoskeleton with phalloidine (49409-10NMOL, Sigma-Aldrich, USA, 5 μg/ml) and observed with confocal microscopy (FV1000, Olympus, Tokyo, Japan).

*Immunofluorescence staining of BMDMs*

The time-course expression and localization of BMDMs was assessed by immunofluorescence staining. After the designated treatment, the BMDMs were washed using PBS before fixed by 4% paraformaldehyde and then blocked with goat serum before individually incubated with inducible nitric oxide synthase (iNOS) (ab15323, Abcam) and CD206 (ab64693, Abcam). Then the BMDMs were incubated with secondary antibody (US Everbright Inc., Suzhou, China). After incubation, the sections were washed with phosphate-buffered saline and the nuclei were counterstained with DAPI (Invitrogen). The immunofluorescence images were taken with the confocal microscope (FV1000, Olympus, Tokyo, Japan). Relative fluorescence intensity analysis were analyzed with Image-pro Plus 6.0 (Media Cybernetics, Rockville, MD).

*Live-cell imaging*

The cells were washed 3 times with Hank’s balanced salt solution. The stained cells were washed with Hank’s balanced salt solution and Live Cell Imaging Solution (Thermo Fisher Scientific). Live-cell imaging was performed with the confocal scanning laser microscopy (CLSM; Leica Microsystems, Wetzlar, Germany) according to the manufacturer’s instruction.

*Immuno-TEM*

Immuno-TEM was used to identify the Annexin-positive apoVs. After fixed in 2.5% glutaraldehyde, fibroblasts cultured in collagen hydrogel were blocked with bovine serum albumin (MilliporeSigma, Burlington, MA, USA), and then incubated with mouse anti-Annexin A2 primary antibodies (ab189473, Abcam) and anti-Annexin A4 primary antibodies (ab247624, Abcam). After incubated with gold-conjugated secondary antibodies (1.4 nm nanogold; Nanoprobes, Yaphank, NY, USA; 1:1500 dilution) and silver-enhanced with HQ Silver (Nanoprobes), the specimens were then immersed in osmic acid, dehydrated using an ascending ethanol series, immersed in propylene oxide, and embedded in epoxy resin. Sections (90 nm thick) were stained with uranyl acetate and lead citrate, and observed using a JEM-123 transmission electron microscope (TEM, JEOL, Tokyo, Japan).

**References**

Aran D., Looney A.-P., Liu L., Wu E., Fong V., Hsu A., Chak S., Naikawadi R.-P., Wolters P.-J., Abate A.-R., Butte A.-J. (2019). Bhattacharya M. Reference-based analysis of lung single-cell sequencing reveals a transitional profibrotic macrophage. *Nature immunology*, 20(2),163-172.

Blondel V.-D., Guillaume J.-L., Lambiotte R., Lefebvre E. (2008) Fast unfolding of communities in large networks. *Journal of statistical mechanics: theory and experiment*, 2008(10), P10008.

Maaten L., Hinton G. (2008) Visualizing data using t-SNE. *Journal of machine learning research*, 9, 2579-2605.

Satija R., Farrell J.-A., Gennert D., Schier A.-F., Regev A. (2015). Effects of hypoxia on the proliferation, mineralization and ultrastructure of human periodontal ligament fibroblasts in vitro. *Nature biotechnology*, 33(5), 495-502.

Stuart T., Butler A., Hoffman P., Hafemeister C., Papalexi E., Mauck W.-M., Hao Y., Stoeckius M., Smibert P., Satija R. (2019). Comprehensive Integration of Single-Cell Data. *Cell*, 177(7), 1888-1902.

Xu X., Zhang Y., Wang X., Li S., & Tang L. (2021). Substrate Stiffness Drives Epithelial to Mesenchymal Transition and Proliferation through the NEAT1-Wnt/β-Catenin Pathway in Liver Cancer. *International Journal of Molecular Sciences*, 22, 12066.

Zhang W., Wang G., Liu Y., Zhao X., Zou D., Zhu C., Jin Y., Huang Q., Sun J., Liu X., Jiang X., Zreiqat H. (2013). The synergistic effect of hierarchical micro/nano-topography and bioactive ions for enhanced osseointegration. *Biomaterials*, 34(13), 3184-3195.
